# Imprints of somatic hypermutation on B-cell receptor immunoglobulins post-infection versus post-vaccination against SARS-CoV-2

**DOI:** 10.1093/immhor/vlaf021

**Published:** 2025-06-09

**Authors:** Elisavet Vlachonikola, Nikolaos Pechlivanis, Georgios Karakatsoulis, Massimo Degano, Fotis Psomopoulos, Andrea Crisanti, Giovanni Tonon, Paolo Ghia, Kostas Stamatopoulos, Enrico Lavezzo, Anastasia Chatzidimitriou

**Affiliations:** Institute of Applied Biosciences, Centre for Research and Technology Hellas, Thessaloniki, Greece; Institute of Applied Biosciences, Centre for Research and Technology Hellas, Thessaloniki, Greece; Department of Genetics, Development and Molecular Biology, School of Biology, Aristotle University of Thessaloniki, Thessaloniki, Greece; Institute of Applied Biosciences, Centre for Research and Technology Hellas, Thessaloniki, Greece; Biocrystallography Unit, Division of Immunology, Transplantation, and Infectious Diseases, IRCCS Scientific Institute San Raffaele, Milan, Italy; Università Vita-Salute San Raffaele, Milan, Italy; Institute of Applied Biosciences, Centre for Research and Technology Hellas, Thessaloniki, Greece; Department of Molecular Medicine, University of Padova, Padova, Italy; Università Vita-Salute San Raffaele, Milan, Italy; Center for Omics Sciences, IRCCS Ospedale San Raffaele, Milan, Italy; Università Vita-Salute San Raffaele, Milan, Italy; Division of Experimental Oncology, IRCCS Ospedale San Raffaele, Milan, Italy; Institute of Applied Biosciences, Centre for Research and Technology Hellas, Thessaloniki, Greece; Department of Molecular Medicine and Surgery, Karolinska Institutet, Stockholm, Sweden; Department of Molecular Medicine, University of Padova, Padova, Italy; Institute of Applied Biosciences, Centre for Research and Technology Hellas, Thessaloniki, Greece; Department of Molecular Medicine and Surgery, Karolinska Institutet, Stockholm, Sweden

**Keywords:** adaptive immunity, B-cell receptor gene repertoire, COVID-19, somatic hypermutation

## Abstract

Published evidence supports significant heterogeneity of immune responses among individuals infected with or vaccinated against SARS-CoV-2. This highlights the need for in-depth investigation of the implicated processes toward refined understanding and improved management of COVID-19. The main objective of the present study was to investigate the dynamics of B cell responses to SARS-CoV-2, focusing on how initial infection and subsequent vaccination influence the immunoglobulin gene repertoire, with special emphasis on the impact of somatic hypermutation (SHM) on antibody maturation. Samples were collected from 81 individuals infected by SARS-CoV-2 in the municipality of Vo' during the first pandemic wave in 2020. For 25 of them, sampling was repeated 7 d after completing the primary vaccination series. Deep immunogenetic analysis of the B-cell receptor immunoglobulin (BcR IG) gene repertoire was performed using targeted next-generation sequencing. Bioinformatics analysis focused on repertoire metrics, prediction of IG antigen specificity, and detailed profiling of the SHM patterns. Significant expansions of unmutated sequences early post-infection suggest extrafollicular B cell maturation. In contrast, vaccination promoted SHM acquisition, indicating a germinal center–dependent response, and pronounced repertoire renewal. Restricted SHMs in SARS-homologous clonotypes along with preferential targeting of specific codons within the VH domain post-vaccination support ongoing affinity maturation within germinal centers. Differences in the BcR IG profiles post-infection versus post-vaccination allude to distinct trajectories in B cell maturation. Distinct profiles of SHM targeting reflect ongoing affinity maturation post-vaccination, with implications for optimizing preventive and therapeutic interventions against COVID-19.

## Introduction

Immune responses in patients with COVID-19 show significant heterogeneity, likely reflected in diverse clinical manifestations ranging from no/mild symptoms to severe respiratory failure and multiorgan failure leading to death.^1^ Adding to this complexity, varying levels of acquired protection from vaccination have also been observed.^2^ This underscores the need to fully understand the immune mechanisms underlying these diverse outcomes in order to improve the management of COVID-19 and enhance immunization strategies against SARS-CoV-2.

Recent studies have outlined distinct trajectories in B-cell maturation and differentiation upon SARS-CoV-2 infection. Evidence suggests that a primary SARS-CoV-2 infection bypasses the normal germinal center (GC) reaction, which typically generates long-lived plasma cells, favoring instead an extrafollicular reaction in which B cells rapidly proliferate and differentiate into short-lived plasma cells.^3^ Contradictory results have been reported regarding the neutralizing capacity and affinity of these early-secreted antibodies.^4^^,^^5^ Moreover, the molecular imprints of extrafollicular maturation on the B-cell receptor immunoglobulin (BcR IG) gene repertoire have not been conclusively determined.^3^^,^^6^

Early observations on the declining neutralizing antibody responses within the first year post-infection and the role of memory B-cell responses for stronger and longer-lasting protection during reinfection supported the design of vaccination strategies.^7^ However, the relatively short period of time since the onset of the SARS-CoV-2 pandemic precludes definitive conclusions from being drawn regarding the optimal protection strategies. This explains why the debate continues on whether vaccinating previously infected individuals could further reduce the risk of reinfection and transmission. Therefore, understanding the temporal dynamics and the B-cell response molecular landscape both post-infection and post-vaccination is crucial for the future design of more effective immunization strategies, toward a better long-term control of COVID-19.

Here, utilizing next-generation sequencing (NGS), we investigated the BcR IG gene repertoire profiles in a unique cohort of cases infected by SARS-CoV-2 during the first pandemic wave in spring 2020 and subsequently vaccinated against the virus.^8^ We focused on clonality patterns, immunoglobulin gene usage, and SARS-CoV-2–biased somatic hypermutation (SHM) signatures after the first encounter with SARS-CoV-2 and a subsequent round of vaccination. We report the existence of expanded clonotypes with minimal/no SHM post-infection, indicative of extrafollicular maturation. Moreover, we show that post-infection vaccination against SARS-CoV-2 following a prior infection caused a significant renewal of the BcR IG gene repertoire. Notably, post-vaccination SARS-homologous BcR IG clonotypes were predominantly mutated, showed preferential usage of particular IGHV genes, and carried restricted replacement SHMs, indicative of ongoing affinity maturation in germinal centers. These findings emphasize the dynamic interplay between SHM and immune responses, highlighting the impact of vaccination on SARS-specific B-cell repertoires.

## Materials and methods

### Patients and samples

Samples were collected as part of an epidemiological study investigating the prevalence of SARS-CoV-2 infection in the municipality of Vo', located in the province of Padua, Veneto region, Italy, during February to May 2020.^8^ During this period, peripheral blood samples and nasopharyngeal specimens were obtained from the respiratory tract of 81 individuals who were either hospitalized or tested positive for COVID-19 within screening programs. The detection of SARS-CoV-2–positive individuals was performed using quantitative polymerase chain reaction (PCR). Anti-SARS-COV-2 antibody response was assessed through 3 commercial serology testing kits: LIAISON SARS-CoV-2 S1/S2 IgG (DiaSorin Molecular), Elecsys Anti-SARS-CoV-2 assay (Roche Diagnostics), and ARCHITECT SARS-CoV-2 IgG (Abbott Laboratories).^9^ Manufacturer cut-offs were used for determining if samples were positive by each test.

Sampling was repeated in 25 individuals of the study cohort in June 2021, at least 7d after vaccination with either the Pfizer-BioNTech or the Oxford-AstraZeneca COVID-19 vaccine.^10^ Participants provided written informed consent before sampling, and all data used in this study were previously pseudonymized. The study was approved by the Ethics Committee for Clinical Research of the province of Padova (protocol numbers 0026971 and 0068830) and conducted in compliance with the ethical principles for medical research stated in the Declaration of Helsinki. A detailed description of the study cohort is given in Table S1.

**Table 1.** Significant amino acid substitutions observed in SARS-homologous clonotypes following infection or vaccination against SARS-CoV-2.

Gene	Position		Reference	Alteration	Region	*P* value		Type of alteration	Hotspot
**Post-infection repertoire**									
**IGHV3-30**	36		S	N	CDR1	0.009		Non-conservative	Y
**IGHV3-30**	64		N	K	CDR2	0.011		Non-conservative	Y
**IGHV3-30**	64		N	T	CDR2	0.022		Non-conservative	Y
**IGHV3-30**	40		H	Y	FR2	0.046		Non-conservative	N
**IGHV3-30**	97		E	D	FR3	0.029		Conservative	N
**IGHV3-30**	86		T	S	FR3	0.033		Conservative	N
**IGHV3-33**	86		T	S	FR3	0.010		Conservative	N
**Post-vaccination repertoire**									
**IGHV3-11**	65		I	K	CDR2	0.032		Non-conservative	Y
**IGHV3-11**	86		S	T	FR3	0.042		Conservative	N
**IGHV3-21**	14		K	T	FR1	0.034		Non-conservative	Y
**IGHV3-21**	38		S	T	CDR1	0.031		Conservative	Y
**IGHV3-30**	51		E	D	FR2	0.007		Conservative	N
**IGHV3-30**	55		V	A	FR2	0.048		Conservative	Y
**IGHV3-30**	63		S	L	CDR2	0.00004		Non-conservative	Y
**IGHV3-30**	68		A	T	FR3	0.046		Non-conservative	N
**IGHV3-30**	69		D	T	FR3	0.00008		Non-conservative	N
**IGHV3-30**	77		T	S	FR3	0.00001		Conservative	N
**IGHV3-30**	84		K	N	FR3	0.00002		Non-conservative	N
**IGHV3-30-3**	3		Q	R	FR1	0.032		Non-conservative	N
**IGHV3-30-3**	27		G	E	CDR1	0.029		Non-conservative	N
**IGHV3-30-3**	72		K	R	FR3	0.003		Conservative	Y
**IGHV3-30-3**	103		Y	F	FR3	0.019		Conservative	Y
**IGHV3-33**	24		A	S	FR1	0.038		Non-conservative	N
**IGHV3-33**	36		S	N	CDR1	0.020		Non-conservative	Y
**IGHV3-33**	55		V	A	FR2	0.034		Conservative	Y
**IGHV3-33**	56		I	V	CDR2	0.040		Conservative	Y
**IGHV3-33**	72		K	E	FR3	0.037		Non-conservative	N
**IGHV3-48**	38		S	Y	CDR1	0.047		Non-conservative	Y
**IGHV3-48**	65		I	K	CDR2	0.012		Non-conservative	Y
**IGHV3-48**	91		M	L	FR3	0.048		Non-conservative	Y
**IGHV3-66**	38		Y	D	CDR1	0.017		Non-conservative	Y
**IGHV3-66**	64		S	I	CDR2	0.021		Non-conservative	N
**IGHV3-66**	84		K	R	FR3	0.023		Conservative	Y
**IGHV3-66**	103		Y	F	FR3	0.022		Conservative	Y
**IGHV3-74**	56		I	M	CDR2	0.048		Non-conservative	Y
**IGHV3-74**	58		S	T	CDR2	0.003		Conservative	N
**IGHV3-74**	96		A	T	FR3	0.045		Non-conservative	N

The term *conservative* denotes substitutions resulting in the replacement of an amino acid by another one possessing similar physicochemical properties, as defined in standardized classes used in IMGT databases.

N, no; Y, yes.

### NGS of IGHV-IGHD-IGHJ gene rearrangements

BcR IG gene rearrangements were amplified on genomic DNA isolated from peripheral blood mononuclear cells (PBMCs). PCR amplification of IGHV-IGHD-IGHJ gene rearrangements was performed by Platinum Taq DNA Polymerase High Fidelity (Thermo Fisher Scientific) using IGHV subgroup specific primers annealing to the leader sequence and IGHJ gene primers.^11^ Amplicons were purified by excising the PCR product from an agarose gel, followed by gel extraction (Qiagen). Library construction was performed according to the manufacturer’s instructions (NEB Next Ultra II DNA Library Prep Kit for Illumina), and NGS was performed with the MiSeq Reagent Kit v3 (2 × 300 bp) on the MiSeq Benchtop Sequencer (Illumina).^12^

Paired-end read merging and strict length/quality filtering were performed by a purpose-built bioinformatics pipeline.^13^ Annotation of the IGHV-IGHD-IGHJ gene rearrangements was carried out by the IMGT/HighV-QUEST tool^14^ and final meta-data analysis was performed using the TRIP (T cell Receptor/Immunoglobulin Profiler) tool.^15^

For the interpretation of the NGS results, the term *clonotype* was used to describe IGHV-IGHD-IGHJ gene rearrangements with unique pairs of IGHV genes and identical heavy variable complementarity determining region 3 (VH CDR3) amino acid sequences within a sample. The 10 most frequent clonotypes within a sample are herein referred to as “major.”. he relative frequency of each clonotype in a given sample was calculated as the number of IGHV-IGHD-IGHJ gene rearrangements corresponding to this particular clonotype divided by the total number of productive, filtered-in IGHV-IGHD-IGHJ gene rearrangements of that sample. Repertoire clonality was estimated as the median cumulative frequency (MCF-10) of the major clonotypes. In addition, in order to evaluate the relative frequency of each IGHV gene that partakes in the clonotype formation, the number of clonotypes using particular IGHV genes was calculated over the total number of clonotypes.

### Prediction of SARS-homologous BcR IG clonotypes

To predict the SARS-homologous BcR IG clonotypes in the cohort, all published/patented antibodies (9,162 entries) able to bind to SARS-CoV-2 deposited in CoV-AbDab were used to calculate sequence similarities.^16^ As a first step, we computed the length of the CDR3 region of all BcR IG sequences between the cohort and database. Subsequently, for CDR3 sequences with identical lengths, we calculated the Hamming distance to gauge their dissimilarity, and distances falling below 85% amino acid sequence similarity were filtered out. The similarity score was calculated following the standardized classes used in IMGT databases, allowing amino acids with common physicochemical properties to be clustered together.^17^ BcR IG clonotypes sharing rearrangements of the same IGHV gene subgroup and displaying CDR3 amino acid sequence similarity >85% with a particular CoV-AbDab entry were designated as SARS-homologous.

### Mapping of SHM on BcR IG structures

The structures of representative fragment antigen-binding region (Fab) and single-chain fragment variable (scFv) fragments utilizing certain IGHV genes were downloaded from the RCSB Protein Data Bank (RCSB PDB) (http://www.rcsb.org)^18^ and visualized using Pymol v1.8 (http://www.pymol.org). The associated PDB codes were 3FZU, 6XKP, and 7CDI (IGHV3-11); 7L09 and 5IFH (IGHV3-21); 6XYZ (IGHV3-30 and IGHV-30-3); 7LXW (IGHV3-33); 6ZTD (IGHV3-48); 3B9V (IGHV3-66); and 4UT7 (IGHV3-74). Intermolecular contacts within the deposited structures were analyzed using PISA.^19^ Mutations were modeled using the mutation module in Pymol without further energetic optimization.

### Statistical analysis

For the descriptive statistics, frequencies and relative frequencies were used for the categorical variables, with median (interquartile range) for the numeric variables. To compare the clonotype relative frequencies between the mutated and unmutated cases, and between the post-infection and post-vaccination cases, the binomial test was used. The Wilcoxon test was applied to investigating potential differences in the repertoire between the SARS-homologous and non-homologous clonotypes within rearrangements utilizing a specific IGHV gene or carrying CDR3s of identical length. Last, Fisher's exact test was applied for the comparison of relative IGHV gene frequencies in post-infection versus post-vaccination cases. The significance level was set to 5%, and all *P* values were exploratory. To correct for the multiplicity issues, the false discovery rate correction was used. All the analyses were conducted in R (v. 4.3.2; R Foundation for Statistical computing).

## Results

### Distinct BcR IG gene repertoire profiles post-infection versus post-vaccination

**Figure 1.**
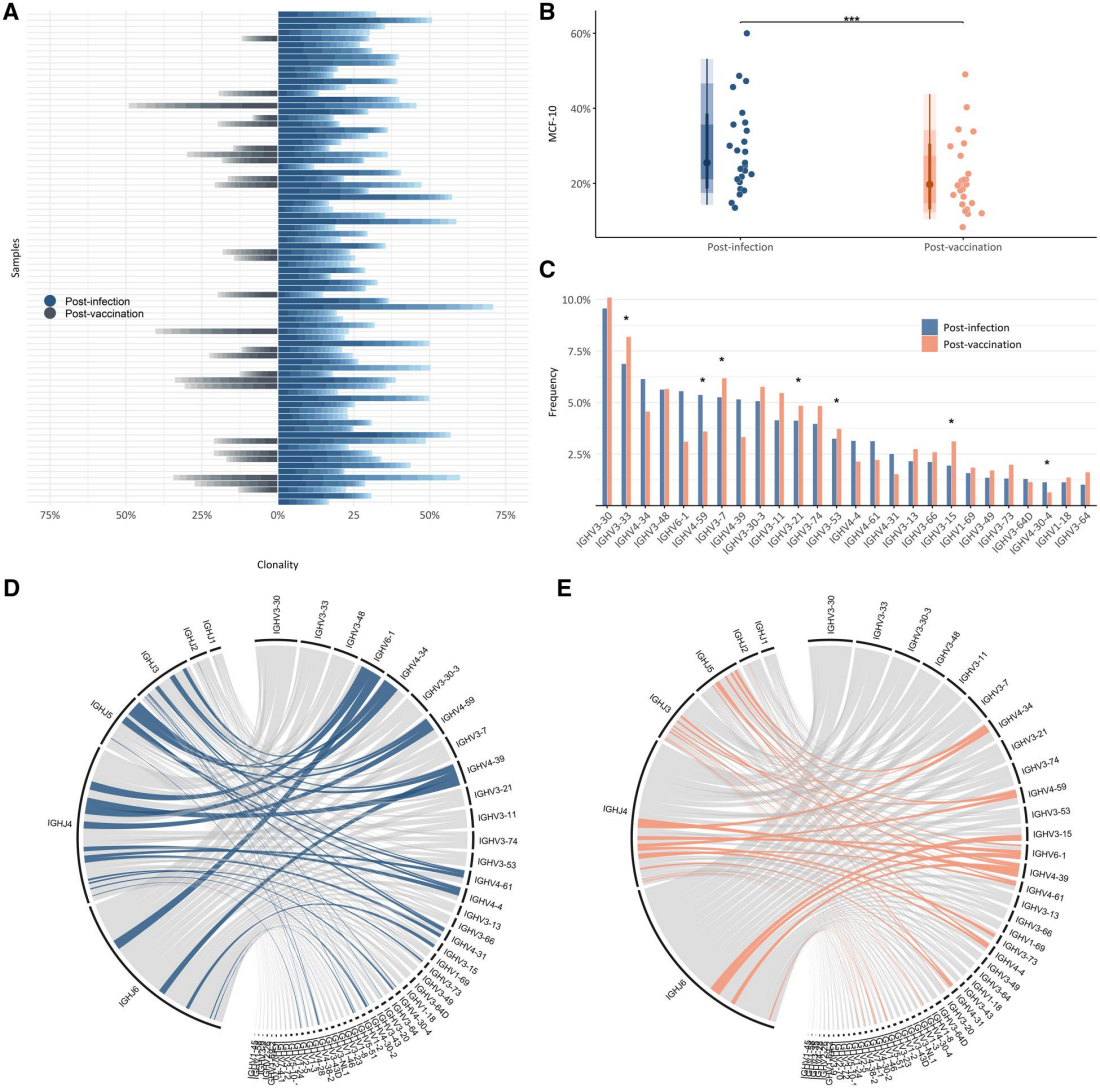
Comparison of BcR IG gene repertoire features post-infection versus post-vaccination. (A) Bar plots depict the frequency of the 10 immunodominant (major) clonotypes in the post-infection and post-vaccination BcR IG gene repertoires, emphasizing the oligoclonal profile of the post-infection repertoire. (B) Density box plots show the distribution of clonality across paired samples at the two time points: clonality was found to be increased post-infection versus post-vaccination (*P* = 0.0007). Clonality was estimated as the MCF-10 and is represented as points, indicating clonality per sample. (C) Bar plots illustrate the frequency of the utilized IGHV genes, revealing differences between the two time points (*P* < 0.05). (D) Circos diagrams depict IGHV-IGHJ gene rearrangements at the two time points, with colored connections indicating statistically significant signatures (***P < 0.05).

In total, 5,267,301 annotated reads were filtered-in and analyzed as productive gene rearrangements, resulting in the identification of 357,252 BcR IG clonotypes (i.e. IGHV-IGHD-IGHJ gene rearrangements with unique pairs of IGHV genes and identical heavy VH CDR3 amino acid sequences within a sample) in all individuals at the 2 sampling time points (median number of clonotypes per sample: 2,996 post-infection and 2,760 post-vaccination). Pairwise comparisons of the post-infection versus post-vaccination BcR IG gene repertoires in 25 individuals with available longitudinal samples revealed similar repertoire diversity at the two time points (median Shannon index: 5.86 post-infection versus 5.68 post-vaccination). Although oligoclonal expansions were evident in both subgroups (Fig. 1A), pairwise comparisons revealed significantly (*P* < 0.0007) higher clonality in the post-infection (estimated as the median cumulative frequency of the 10 major clonotypes of each sample: MCF-10, 29.02% [range: 11.92%–70.87%]) versus the post-vaccination samples (MCF-10, 19.72% [range: 8.34%–49.03%]) (Fig. 1B). Moreover, differences in the frequency of individual IGHV genes were documented in the repertoires of the two time points, with the IGHV3-33, IGHV3-7, IGHV3-21, IGHV3-53, and IGH3-15 genes showing increased frequency post-vaccination (*P* < 0.05) (Fig. 1C). Recombination events between the IGHV3-30 and IGHJ6 genes were among the most frequent at both time points. That said, other IGHV-IGHJ gene rearrangements were significantly more frequent at each time point (*P* < 0.05) (Fig. 1D). Notably, the BcR IG gene repertoires appeared to be completely renewed post-vaccination, with only 302 (0.35%) of 86,067 clonotypes retained over time by 15 of 25 vaccinated individuals.

### Different SHM signatures post-infection versus post-vaccination

For the analysis of the SHM status of the BcR IG repertoires, we categorized BcR IG clonotypes into distinct subgroups: (i) those bearing IGHV genes with <100% identity compared with the corresponding IGHV gene and allele germline sequence, which we refer to as mutated, and (ii) those displaying no SHM on the rearranged IGHV gene (i.e. displaying 100% IGHV gene and allele germline identity), which we refer to as unmutated.

**Figure 2.**
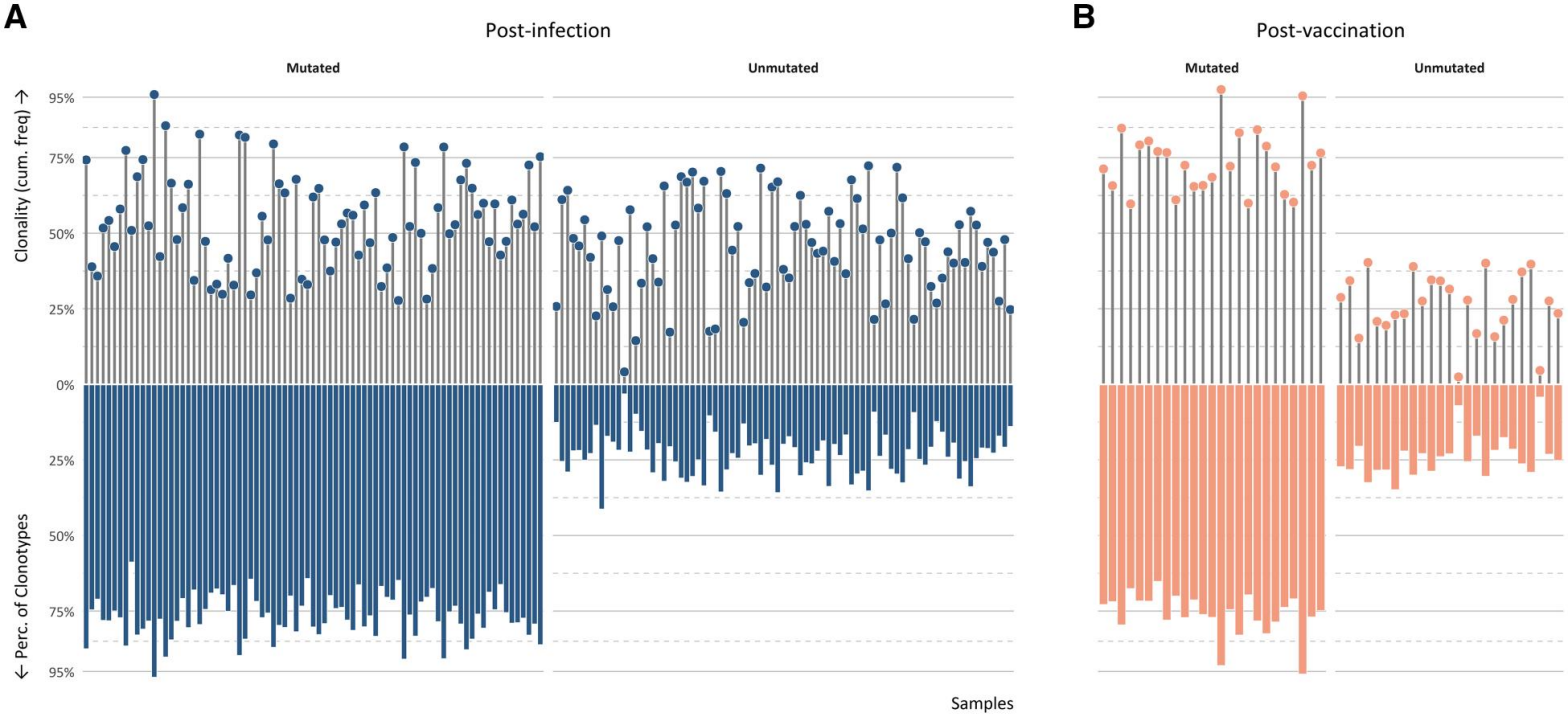
SHM status and clonality. Lollipop graphs depict clonality, showing cumulative frequencies of mutated and unmutated clonotypes per sample at the two time points. Bar plots illustrate the percentage of mutated and unmutated clonotypes per sample. The binomial test was employed to assess potential differences in the fraction occupied by the mutated and unmutated clonotypes in post-infection versus post-vaccination repertoires, revealing statistically significant results (*P* < 0.0001) between the two time points.

In the post-infection samples, the majority of clonotypes (median: 78.27% [range: 64.21%–96.88%]) in all samples were classified as mutated. That notwithstanding, while unmutated clonotypes constituted a smaller fraction of the repertoire of unique clonotypes (median: 21.73% [range: 3.12%–35.79%]), they tended to be larger in size (MCF of unmutated clonotypes: 42% [range: 4.2%–72.3%] vs MCF of mutated clonotypes: 57.9% [range: 27.7%–95.8%]) (Fig. 2A).

Predominance of mutated clonotypes was also noted in the post-vaccination samples (median: 74.84% [range: 64.17%–95.82%]). However, these samples included significantly less expanded unmutated clonotypes compared with the post-infection samples (MCF: 27.56% post-vaccination vs 42% post-infection; *P* < 0.0001) (Fig. 2B).

### Immunogenetic characterization of SARS-homologous clonotypes

**Figure 3.**
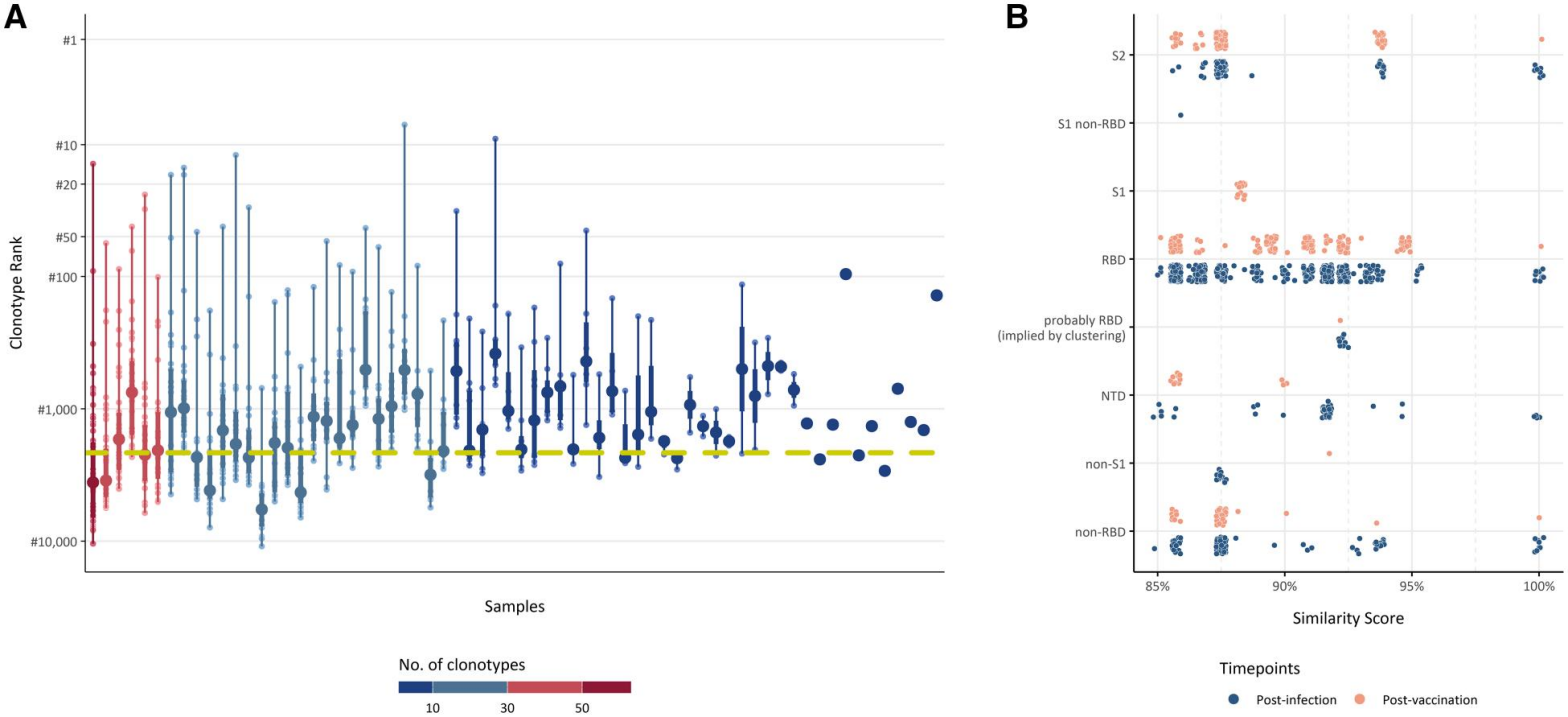
SARS-homologous clonotypes. (A) Density box plots illustrate the distributions of post-infection BcR IG gene clonotypes binding to SARS-derived epitopes. The colors represent the number of clonotypes, and the density indicates the percentage of values, with the darkest region encompassing 50% of the observations. (B) Points represent clonotypes that exhibit more than 85% similarity to known neutralizing antibodies in CDR3 amino acid sequences. The colors indicate different time points.

*In silico* prediction of the specificity of BcR IG clonotypes resulted in the identification of 764 unique SARS-homologous clonotypes within the BcR IG gene repertoires of 66 (81.5%) of 81 individuals who tested positive for COVID-19 during the first pandemic wave (Table S2). SARS-homologous clonotypes were identified with varying frequencies, and, among them, 26 (3.4%) ranked in the 100 most frequent clonotypes (Fig. 3A). Additionally, 276 SARS-homologous clonotypes were identified in the BcR IG gene repertoires of 21 (84%) of 25 vaccinated individuals, reflecting a comparable clonotype distribution (3.6% ranked in the 100 most frequent clonotypes). The majority of SARS-homologous clonotypes, specifically 70.5% of post-infection and 45.1% of post-vaccination clonotypes, exhibited a high similarity index to neutralizing antibodies targeting epitopes within the receptor-binding domain of the SARS-CoV-2 spike protein (Fig. 3B). Notably, 70 (9.16%) of 764 post-infection SARS-homologous clonotypes showed a high degree of similarity to therapeutic antibodies developed by Regeneron, derived either from humanized mice or convalescent patients. In sharp contrast, only 5 (1.8%) of 276 post-vaccination SARS-homologous clonotypes, found in different individuals than those post-infection cases, resembled known therapeutic antibodies (Table S3).

#### IG gene repertoire

Analysis of the SARS-homologous clonotypes post-infection revealed a strong preference for the usage of particular IGHV genes compared with the non–SARS-homologous clonotypes. In particular, SARS-homologous clonotypes showed enrichment for rearrangements of the IGHV3 gene subgroup, while the remaining clonotypes exhibited a higher prevalence of IGHV4 gene rearrangements (*P* < 0.05). Of note, SARS-homologous clonotypes post-vaccination showed biased usage of the IGHV3-30 gene (median frequency: 22% in SARS-homologous vs 10.19% in the remaining clonotypes; *P* = 0.0002).

#### SHM targeting

**Figure 4.**
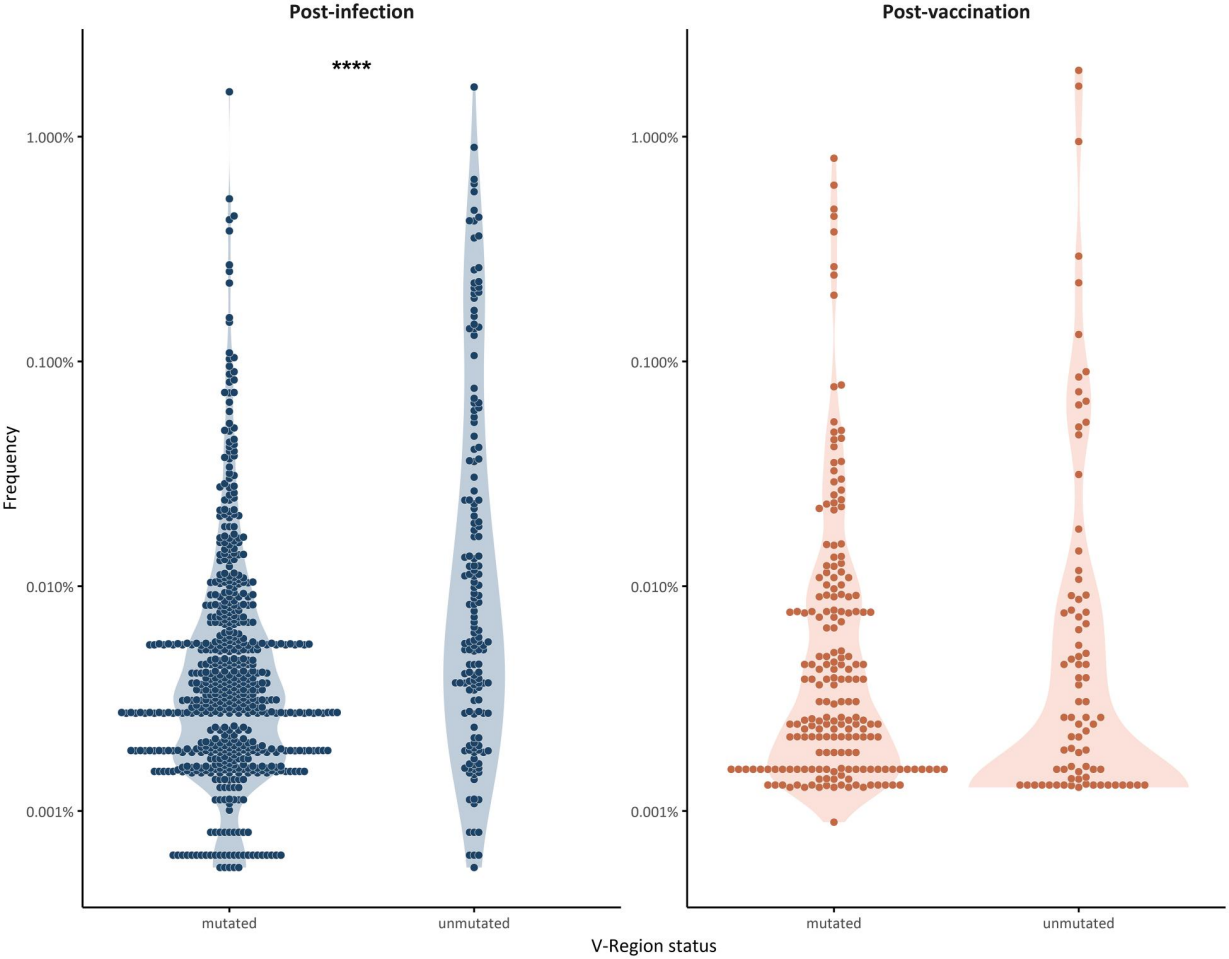
Frequency of SARS-homologous clonotypes with respect to SHM status. Points depict the frequency of all mutated and unmutated SARS-homologous clonotypes for the paired samples at (A) post-infection versus (B) post-vaccination, revealing a statistically significant expansion of the unmutated clonotypes post-infection (*P* = 0.0002).

A total of 604 (79%) of 769 SARS-homologous clonotypes post-infection were mutated, whereas the remaining 165 (21%) were unmutated. In line with our observations in the entire repertoire, unmutated clonotypes, though fewer in number, were often considerably expanded, thus differing significantly (*P* = 0.0002) (Fig. 4A) from mutated clonotypes, which were predominantly small. Similar to what we found in the entire repertoire, no statistically significant correlation was observed between SHM status and clonal size among post-vaccination SARS-homologous clonotypes (Fig. 4B).

Next, we characterized in detail replacement SHMs toward obtaining hints regarding their potential relevance for shaping the binding affinity of the BcR IG clonotypes to SARS-related antigens. When comparing amino acid replacements per position in different SARS-homologous clonotypes that utilized the same IGHV gene, we found that, in many cases, distinct SHMs convergently encoded a particular conserved amino acid substitution. Furthermore, comparisons between post-infection versus post-vaccination SARS-homologous repertoires revealed biases in SHM topology within rearrangements utilizing particular IGHV genes. Specific codons within the VH domain were preferentially targeted at each time point, as shown in Table 1.

Detailed analysis of the topology of nucleotide mutations generated by SHM, and the resulting amino acid substitution profiles, revealed that not all observed SHMs occurred in hotspots. Instead, they were equally distributed throughout the variable domain with no significant differences between post-infection versus post-vaccination repertoires. However, in the post-vaccination repertoires we observed a statistically significant enrichment for replacement SHMs at particular positions within the variable domain (Table 1). In 60% of cases, these replacements led to the introduction of an amino acid residue acid with different physicochemical properties compared with the germline-encoded residue at this particular position.

**Figure 5.**
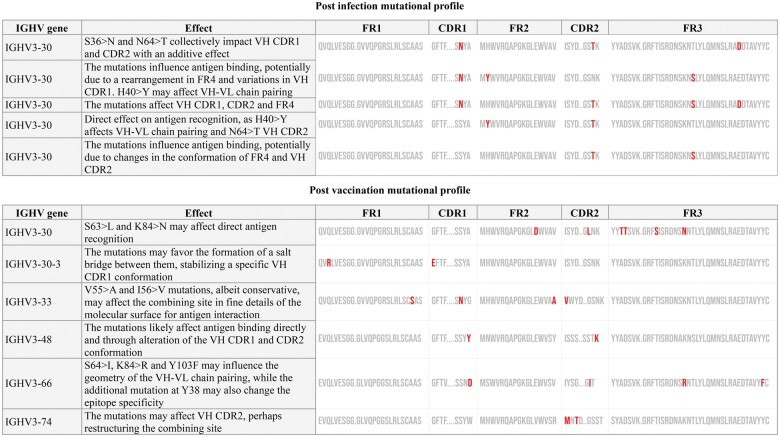
Significant amino acid substitutions observed in SARS-homologous clonotypes as a string of co-existing SHMs in rearrangements of a particular IGHV gene.

Next, we sought to assess *in silico* the potential relevance of the observed amino acid substitutions in each SARS-homologous clonotype. To this end, we took advantage of crystal structures of clonotypes available in the RCSB PDB.^18^ In each case (clonotype) from our study, comparisons of the SHM patterns were made against public (RCSB PDB) clonotypes utilizing the same IGHV. These comparisons highlighted particular SHMs with significant predicted effects on IG conformation, shaping the antigen binding affinity and specificity. In general, non-conservative mutations at IMGT positions VH CDR1 26-38 and VH CDR2 56-65 appeared to critically impact their respective conformation, potentially affecting interactions with antigens. Indicatively, the introduction of G27>E by SHM in SARS-CoV-2 specific clonotypes utilizing the IGHV3-30-3 gene after vaccination alters the physicochemical properties of the wild-type residue, introducing a negatively charged amino acid at the beginning of VH CDR1 (Fig. 5). This alteration appears to influence antigen affinity and may even affect specificity compared with the unmutated clonotype with the germline-encoded sequence.

**Figure 6.**
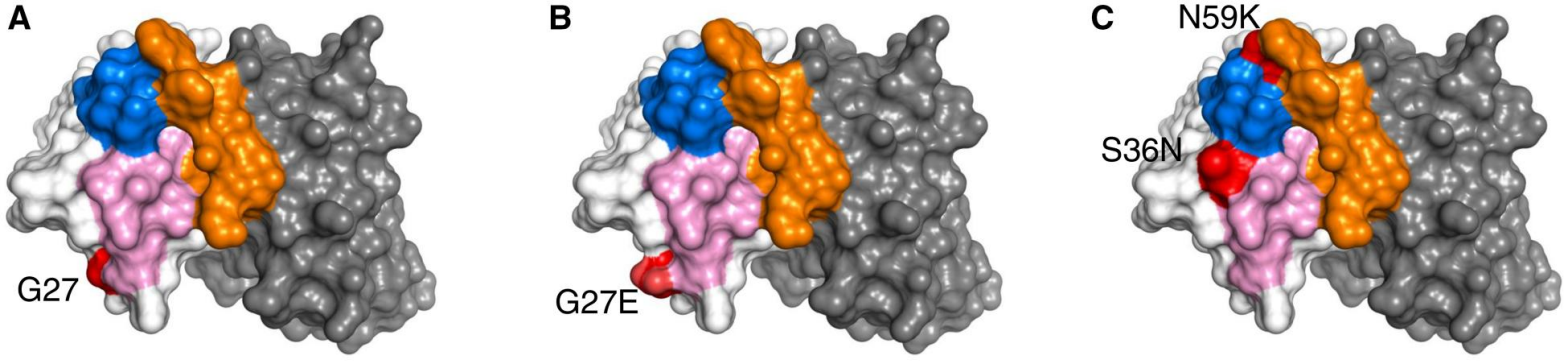
Example of localization of frequent mutations post-vaccination or infection by SARS-CoV-2. The structure of a BcR IG encoded by the IGHV3-30-3 gene (PDB code 6KYZ) is shown through its molecular surface, with the heavy chain in white and the light chain in gray. The HCDR1, HCDR2, and HCDR3 regions are colored pink, blue, and orange, respectively. (A) The position of residue G27 is shown in red. (B) The effect of the mutation to a glutamic acid, a frequent mutation in antibodies from individuals post-vaccination, has a minor effect on the combining site but introduces a negative charge at the edge of the region devoted to antigen binding. (C) The simultaneous introduction of the two Ser36Asn and Asn59Lys mutations observed in antibodies from individuals post-infection affects directly the character of the surface involved in antigen recognition.

Another noteworthy finding concerned the existence of strings of distinct SHMs consistently co-occurring in rearrangements of a particular IGHV gene. These patterns highlighted the additive or synergistic effect of SHMs, while they differed between the post-infection and post-vaccination repertoires, as shown in Fig. 5. Interestingly, post-infection SARS-homologous clonotypes utilizing the IGHV3-30 gene acquired SHMs with a predicted major influence on critical BcR IG domains, anticipated to impact the final IG conformation and antigen recognition (Fig. 6).

**Figure 7.**
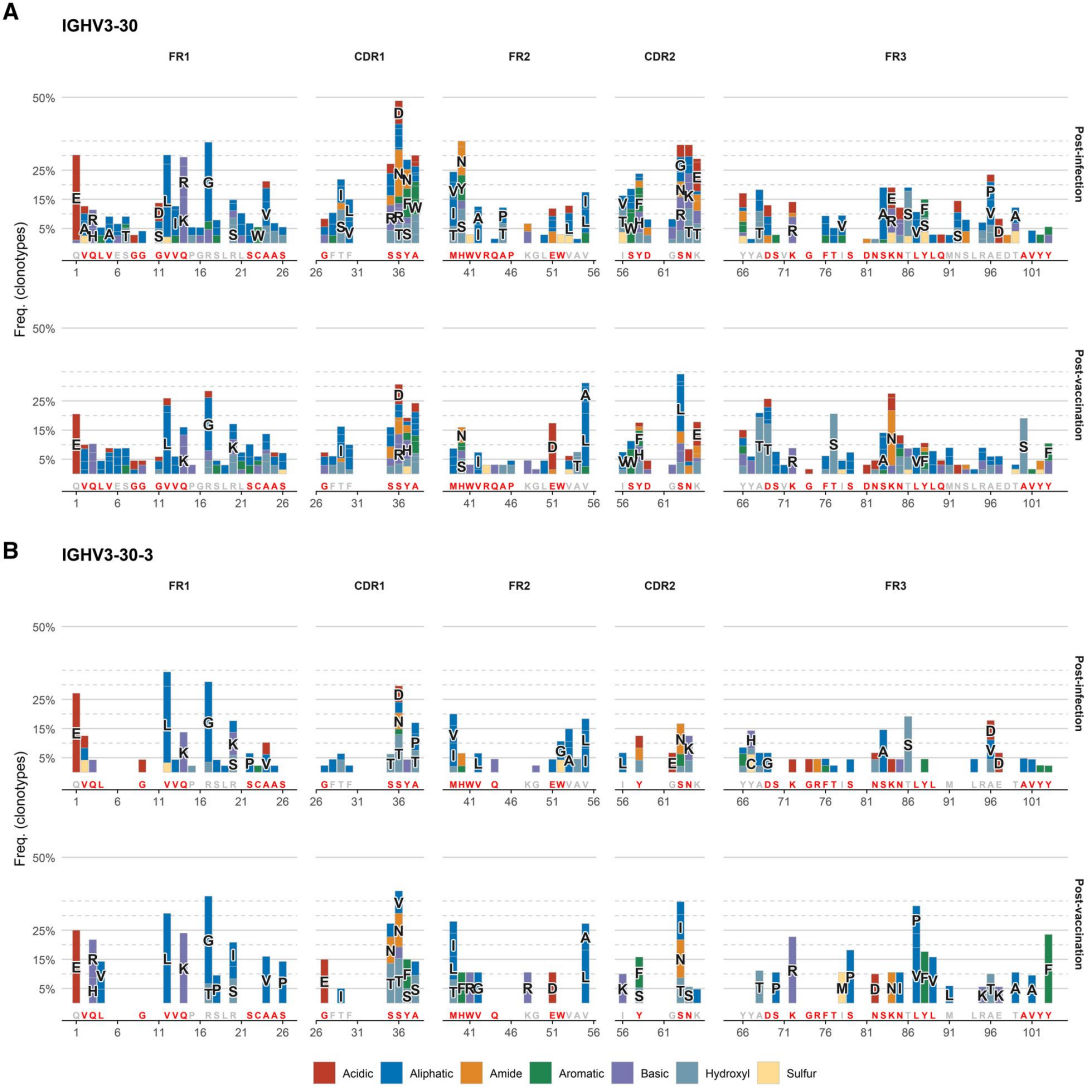
Mutational analysis of SARS-homologous clonotypes. Stacked bar plots illustrate amino acid substitutions resulting from SHM on the FR1-FR3 part of the VH domain of all SARS-homologous IG clonotypes sharing the same IGHV gene in the post-infection and post-vaccination BcR IG gene repertoires. The x-axis letters represent the amino acids encoded by the germline gene sequence and the letters in red depict mutational hotspots, while the named bars indicate amino acid substitutions with a frequency exceeding 5%. For the sake of clarity, SHMs called by a single sequencing read were excluded from the figure. The color code employed to describe the physicochemical properties of each amino acid per position is based on the IMGT database classification: dark blue for aliphatic (A, V, I, L, G, P), yellow for sulfur (C, M), light blue for hydroxyl (S, T), red for acidic (D, E), orange for amide (N, Q), purple for basic (H, K, R), and green for aromatic (F, W, Y) amino acids.

Further comparisons of the amino acid substitution profiles in the BcR IG gene repertoires of individuals analyzed both post-infection as well as post-vaccination revealed two different patterns of SHM acquisition. On the one hand, a particular replacement mutation introduced post-infection increased in frequency post-vaccination (e.g. E51>D and V55>L in SARS-CoV-2 specific clonotypes utilizing the IGHV3-30 gene) (Fig. 7A). On the other hand, certain replacement SHMs introduced post-infection were not detected post-vaccination; instead, the same position in the VH domain was found to carry a different replacement SHM (e.g. positions VH CDR1-27 and VH FR2-51 in SARS-CoV-2 specific clonotypes utilizing the IGHV3-30-3 gene) (Fig. 7B).

## Discussion

In-depth comprehension of the human antibody response to SARS-CoV-2 is a critical prerequisite for advancing effective immune strategies to restrain COVID-19. Previous immunogenetic studies on the B cell antibody response to infection by SARS-CoV-2 suggested a two-tier response, mainly through characterizing patterns of SHM within the IG genes. The identification of near-germline SARS-CoV-2 neutralizing antibodies, exhibiting no or limited degree SHM, was thought as consistent with a rapid maturation of naive B cells through an extrafollicular maturation pathway.^2^ In contrast, studies on the B-cell memory response after the initial infection revealed additional SHMs and clonal expansion over time, consistent with GC maturation.^20^ That said, previous studies on single time points, as well as variations in study designs and cohort composition, hinder solid conclusions on the molecular mechanisms of the B cell response to SARS-CoV-2.

In this study, we profiled the BcR IG gene repertoire after the initial infection by the SARS-CoV-2 virus as well as following a complete primary series of immunization with 2 vaccine doses. A particular strength of the study concerns the fact that the investigated cohort derived from a restricted geographical area in the municipality of Vo', and the sampling was conducted during the first pandemic wave and the initial phase of vaccination strategies,^8–10^ thus offering the opportunity to interrogate the naïve immune responses to a novel antigen.

By in-depth immunogenetic analysis of the BcR IG repertoire, we documented that vaccination against SARS-CoV-2 following the initial infection by the virus resulted in a significant turnover of the BcR IG gene repertoire: of note, clonal expansions detected after the initial infection decreased significantly after vaccination. Responses to vaccination exhibited a bias toward BcR IG clonotypes encoded preferentially by IGHV3 subgroup genes, consistent with previous reports on the spike-specific antibody response induced by vaccination.^21^^,^^22^ Notably, extensive comparisons of post-infection BcR IG repertoire profiles between symptomatic versus asymptomatic cases, hospitalized versus non-hospitalized individuals, and post-vaccination BcR IG repertoires between individuals vaccinated with different vaccine types (Pfizer-BioNTech vs. Oxford-AstraZeneca), did not reveal any statistically significant differences (data not shown). This underscores the distinct effects that initial infection and subsequent vaccination have on shaping the immune repertoire. Analysis of SHM profiles revealed distinct patterns between post-infection versus post-vaccination, which were found to be independent of age distribution in each group. While most clonotypes carried SHMs at both time points, significant expansions of unmutated clonotypes were seen almost exclusively post-infection. This difference held also when focusing specifically on the predicted SARS-homologous clonotypes. These findings reinforce the concept that the initial anti-SARS-CoV-2 immune response following infection predominantly activates extrafollicular B cell maturation, leading to the generation of near-germline anti-SARS-CoV-2 antibodies,^3^^,^^23^ while in contrast, vaccine-induced immunization progressively drives the acquisition of SHM, a hallmark of a canonical GC-dependent immune response^.24^

A notable finding of our study concerns the presence of restricted replacement SHMs in SARS-homologous BcR IG clonotypes utilizing the same IGHV gene, alluding to strong antigen selection that favors the introduction of particular changes in IG structure, likely due to their relevance in anti-viral responses. These findings also suggest a dynamic interplay between SHM profiles and the immune response following vaccination, potentially reflecting ongoing affinity maturation processes and selective pressure exerted by the vaccine antigens.

Comparisons between predicted SARS-homologous versus non-homologous clonotypes highlighted a strong preference for particular IGHV genes in the former, with a notable enrichment for IGHV3 subgroup genes. Vaccination led to a significant further enrichment of SARS-homologous clonotypes utilizing IGHV3 subgroup genes, especially IGHV3-30, indicating a targeted response to vaccine antigens. Nevertheless, these results should be interpreted cautiously due to the inherent limitations of the *in silico* prediction software, which relied on the assessment of previously published or patented antibodies submitted to CoV-AbDab, potentially introducing biases into the analysis.^16^ Future work should focus on generating single-cell BcR IG repertoire data to gain insights into VH and VL pairing in SARS-homologous clonotypes, allowing the generation of monoclonal antibodies from IG sequences for experimental characterization of their antigenic specificity and other physicochemical attributes.

In conclusion, this study underscores the intricate dynamics of the B cell response to SARS-CoV-2, delineating how initial infection and subsequent vaccination uniquely shape the immune repertoire, particularly through the introduction of precisely targeted SHM. Future research should focus on further refining these immunogenetic insights to optimize vaccine design and therapeutic interventions against COVID-19.

## Supplementary Material

vlaf021_Supplementary_Data

## Data Availability

Data is available at the Sequence Read Archive, National Center for Biotechnology Information, under accession number PRJNA1191651. The dataset can be accessed at http://www.ncbi.nlm.nih.gov/bioproject/1191651.
