# Supplementary material for: Imprints of somatic hypermutation on B-cell receptor immunoglobulins post-infection versus post-vaccination against SARS-CoV-2
Source: Immunohorizons. 2025 Jun 9;9(7):vlaf021. doi: 10.1093/immhor/vlaf021 (PMC12148301; doi:10.1093/immhor/vlaf021)
Supplement: vlaf021_Supplementary_Data [file vlaf021_supplementary_data.zip › Supplementary material.pdf]

Supplementary Table 1: An overview of the individuals participated in the study.

Patient ID	Gender	Age group	Post-vaccination Sampling	Symptomatic case	Hospitalized case	Vaccine_type
Pt1	F	61-70	No	Yes	No	NA
Pt2	M	31-40	No	Yes	No	NA
Pt3	F	41-50	No	No	No	NA
Pt4	F	51-60	No	Yes	Yes	NA
Pt5	F	71-80	No	Yes	No	NA
Pt6	F	61-70	No	No	No	NA
Pt7	M	51-60	Yes	Yes	Yes	Pfizer-BioNTech
Pt8	F	31-40	No	Yes	No	NA
Pt9	F	21-30	No	Yes	No	NA
Pt10	M	51-60	Yes	Yes	No	Pfizer-BioNTech
Pt11	F	21-30	No	Yes	No	NA
Pt12	M	61-70	Yes	Yes	Yes	Pfizer-BioNTech
Pt13	M	61-70	Yes	Yes	Yes	Oxford-AstraZeneca
Pt14	M	51-60	No	Yes	No	NA
Pt15	F	31-40	No	Yes	No	NA
Pt16	F	41-50	No	Yes	No	NA
Pt17	M	41-50	No	Yes	No	NA
Pt18	M	71-80	Yes	Yes	No	Oxford-AstraZeneca
Pt19	F	41-50	No	No	No	NA
Pt20	F	41-50	No	No	No	NA
Pt21	M	71-80	No	Yes	No	NA
Pt22	M	21-30	No	Yes	No	NA
Pt23	M	71-80	No	No	No	NA
Pt24	M	51-60	Yes	No	No	Pfizer-BioNTech
Pt25	F	51-60	Yes	Yes	No	Oxford-AstraZeneca
Pt26	F	51-60	No	Yes	No	NA
Pt27	F	61-70	Yes	Yes	No	Pfizer-BioNTech
Pt28	M	31-40	No	Yes	No	NA
Pt29	M	61-70	Yes	No	No	Oxford-AstraZeneca
Pt30	M	71-80	No	No	No	NA
Pt31	M	41-50	No	Yes	Yes	NA
Pt32	F	41-50	No	Yes	No	NA
Pt33	M	21-30	No	No	No	NA
Pt34	M	41-50	No	Yes	No	NA
Pt35	F	71-80	Yes	Yes	No	Pfizer-BioNTech
Pt36	F	31-40	No	Yes	No	NA
Pt37	F	51-60	Yes	Yes	No	Pfizer-BioNTech
Pt38	F	31-40	No	No	No	NA
Pt39	F	51-60	No	Yes	No	NA
Pt40	M	71-80	Yes	Yes	Yes	Pfizer-BioNTech
Pt41	M	31-40	No	Yes	No	NA

Pt42	M	81-90	Yes	Yes	No	Pfizer-BioNTech
Pt43	F	31-40	No	Yes	No	NA
Pt44	F	51-60	No	Yes	No	NA
Pt45	M	21-30	No	Yes	No	NA
Pt46	M	61-70	No	Yes	No	NA
Pt47	F	31-40	No	Yes	No	NA
Pt48	F	71-80	Yes	Yes	No	Pfizer-BioNTech
Pt49	F	61-70	No	Yes	No	NA
Pt50	F	31-40	No	Yes	No	NA
Pt51	M	71-80	No	Yes	No	NA
Pt52	M	71-80	No	No	No	NA
Pt53	M	71-80	No	Yes	Yes	NA
Pt54	M	71-80	Yes	No	No	Pfizer-BioNTech
Pt55	M	31-40	No	No	No	NA
Pt56	M	51-60	No	Yes	No	NA
Pt57	M	51-60	No	Yes	No	NA
Pt58	M	41-50	No	Yes	No	NA
Pt59	M	51-60	No	Yes	No	NA
Pt60	M	51-60	Yes	No	No	Pfizer-BioNTech
Pt61	M	41-50	No	Yes	No	NA
Pt62	F	81-90	Yes	No	No	Pfizer-BioNTech
Pt63	F	61-70	Yes	No	No	Pfizer-BioNTech
Pt64	M	71-80	Yes	No	No	Pfizer-BioNTech
Pt65	M	31-40	No	No	No	NA
Pt66	M	51-60	Yes	Yes	No	Pfizer-BioNTech
Pt67	M	71-80	Yes	No	No	Oxford-AstraZeneca
Pt68	F	51-60	No	No	No	NA
Pt69	M	61-70	Yes	No	No	Pfizer-BioNTech
Pt70	F	31-40	No	No	No	NA
Pt71	F	61-70	Yes	Yes	No	Oxford-AstraZeneca
Pt72	M	61-70	No	No	No	NA
Pt73	M	51-60	No	No	No	Pfizer-BioNTech
Pt74	M	61-70	Yes	Yes	No	Pfizer-BioNTech
Pt75	M	61-70	No	No	No	NA
Pt76	F	61-70	Yes	Yes	No	Oxford-AstraZeneca
Pt77	F	51-60	No	Yes	No	NA
Pt78	M	51-60	No	Yes	No	NA
Pt79	F	41-50	No	Yes	No	NA
Pt80	F	51-60	No	Yes	No	NA
Pt81	F	51-60	No	Yes	No	NA

Supplementary Table 2: A comprehensive list of the identified SARS-specific BcR IG clonotypes per case, along with the matched entry on the CoV-AbDab							
**Patient ID**	**IGHV gene**	**IGH CDR3 (aa)**	**Freq**	**IGHV gene germline identity (%)**	**IGHV gene in db entry**	**IGH CDR3 (aa) in db entry**	**Similarity Score**
Pt1	IGHV3-33	CARDQHLDYW	0.002888	89.58	IGHV3-30-3	CARDQLLDYW	0.90
Pt1	IGHV3-64	CARDQHLDYW	0.002888	94.44	IGHV3-30-3	CARDQLLDYW	0.90
Pt10	IGHV1-69	CARAGYCSGGSCYPLYYYYYGMDVW	0.016632	100	IGHV1-18	CAREGYCSGGSCYSGYYYYYGMDVW	0.88
Pt10	IGHV6-1	CARDPSSFFDYW	0.003326	97.98	IGHV6-1	CARDPSSHFDYW	0.92
Pt10	IGHV3-13	CARDRVRYGMDVW	0.003326	97.54	IGHV3-53	CARDRVVYGMDVW	0.92
Pt10	IGHV1-69	CARGSPYYYDSSGYYGDAFDIW	0.003326	98.61	IGHV1-2	CARGGLYYYDSSAYYGDAFDIW	0.86
Pt11	IGHV3-33	CARDGWYYYYGMDVW	0.001008	98.61	IGHV3-48	CARDGFYYYYAMDVW	0.87
Pt12	IGHV3-7	CARGAYYYYGMDVW	0.001452	100	IGHV3-66	CARGAGYYYGMDVW	0.93
Pt13	IGHV3-23	CARSVSGSYEYYFDYW	0.001374	98.61	IGHV3-30-3	CARSSSGSYLYYFDYW	0.88
Pt14	IGHV1-18	CARDSGGSYSNAAFDIW	0.002389	92.71	IGHV1-46	CARDPGGSYSNDAFDIW	0.88
Pt15	IGHV3-74	CARDLNEYGMDVW	0.003527	94.1	IGHV3-66	CARDLNYYGMDVW	0.92
Pt15	IGHV3-30	CARDLNEYGMDVW	0.031742	94.44	IGHV3-66	CARDLNYYGMDVW	0.92
Pt15	IGHV3-30-3	CARDLNEYGMDVW	0.010581	98.26	IGHV3-66	CARDLNYYGMDVW	0.92
Pt15	IGHV3-33	CARDLNEYGMDVW	0.529026	91.32	IGHV3-66	CARDLNYYGMDVW	0.92
Pt15	IGHV3-7	CARDLNEYGMDVW	0.007054	96.88	IGHV3-66	CARDLNYYGMDVW	0.92
Pt15	IGHV3-11	CARDVGYRVGWFDPW	0.003527	97.57	IGHV3-33	CARDVGSRVTWFDPW	0.87
Pt16	IGHV4-34	CARAHYYYYMDVW	0.001477	96.84	IGHV4-4	CARAYYYYYMDVW	0.92
Pt16	IGHV3-33	CARDGSDYGMDVW	0.001477	100	IGHV3-66	CARDGSAYGMDVW	0.92
Pt16	IGHV3-33	CARDKSDAFDIW	0.00443	98.26	IGHV3-53	CARDISDAFDIW	0.92
Pt16	IGHV3-74	CARDKSDAFDIW	0.002953	97.92	IGHV3-53	CARDISDAFDIW	0.92
Pt16	IGHV3-11	CARDKSDAFDIW	0.001477	92.36	IGHV3-53	CARDISDAFDIW	0.92
Pt16	IGHV3-21	CARDKSDAFDIW	0.001477	98.26	IGHV3-53	CARDISDAFDIW	0.92
Pt16	IGHV3-30	CARDKSDAFDIW	0.001477	92.36	IGHV3-53	CARDISDAFDIW	0.92
Pt16	IGHV3-30-3	CARDKSDAFDIW	0.001477	98.26	IGHV3-53	CARDISDAFDIW	0.92
Pt16	IGHV3-48	CARDKSDAFDIW	0.03987	96.88	IGHV3-53	CARDISDAFDIW	0.92
Pt16	IGHV3-7	CARDKSDAFDIW	0.001477	97.22	IGHV3-53	CARDISDAFDIW	0.92
Pt16	IGHV3-53	CARDLTTAGAFDIW	0.005907	100	IGHV3-53	CARDLAAAGAFDIW	0.86
Pt16	IGHV3-53	CARDLTTAGALDIW	0.001477	99.65	IGHV3-66	CARDLEIAGALDIW	0.86
Pt16	IGHV3-7	CARETAAFDYW	0.010337	91.32	IGHV3-53	CARETLAFDYW	0.91
Pt16	IGHV3-33	CARETAAFDYW	0.002953	98.96	IGHV3-53	CARETLAFDYW	0.91
Pt16	IGHV3-74	CARETAAFDYW	0.001477	94.44	IGHV3-53	CARETLAFDYW	0.91
Pt16	IGHV1-2	CARGVVVPAATPYNWFDPW	0.00886	100	IGHV1-2	CARGWVVPAAIPYNWFDPW	0.89
Pt16	IGHV3-33	CVRGSSYYYYYYMDVW	0.001477	99.31	IGHV3-13	CVRAPSYYYYYYMDVW	0.88
Pt17	IGHV4-61	CAREDYDFWSGYYRGWFDPW	0.000636	95.88	IGHV4-59	CARENYDFWSGYFNGWFDPW	0.85
Pt17	IGHV4-34	CAREDYDFWSGYYRGWFDPW	0.005721	97.54	IGHV4-59	CARENYDFWSGYFNGWFDPW	0.85
Pt17	IGHV4-59	CAREDYDFWSGYYRGWFDPW	0.001907	99.3	IGHV4-59	CARENYDFWSGYFNGWFDPW	0.85
Pt17	IGHV4-39	CAREDYDFWSGYYRGWFDPW	0.018436	100	IGHV4-59	CARENYDFWSGYFNGWFDPW	0.85
Pt17	IGHV3-11	CARELGYYYGMDVW	0.003814	100	IGHV3-66	CARGAGYYYGMDVW	0.86
Pt17	IGHV3-21	CARELGYYYGMDVW	0.000636	97.57	IGHV3-66	CARGAGYYYGMDVW	0.86
Pt17	IGHV3-7	CARELGYYYYGMDVW	0.006993	95.49	IGHV3-74	CAREEGYYYYYMDVW	0.87
Pt17	IGHV3-74	CARELGYYYYGMDVW	0.00445	93.06	IGHV3-74	CAREEGYYYYYMDVW	0.87
Pt17	IGHV3-21	CARELGYYYYGMDVW	0.003814	100	IGHV3-74	CAREEGYYYYYMDVW	0.87
Pt17	IGHV3-33	CARELGYYYYGMDVW	0.003179	96.88	IGHV3-74	CAREEGYYYYYMDVW	0.87
Pt17	IGHV3-13	CARELGYYYYGMDVW	0.001907	99.3	IGHV3-74	CAREEGYYYYYMDVW	0.87
Pt17	IGHV3-53	CARELGYYYYGMDVW	0.001907	100	IGHV3-74	CAREEGYYYYYMDVW	0.87
Pt17	IGHV3-73	CARELGYYYYGMDVW	0.001271	97.96	IGHV3-74	CAREEGYYYYYMDVW	0.87
Pt17	IGHV1-69	CARELGYYYYGMDVW	0.000636	96.88	IGHV1-46	CARELHYYYYYMDVW	0.87
Pt17	IGHV3-48	CARELGYYYYGMDVW	0.028607	98.61	IGHV3-74	CAREEGYYYYYMDVW	0.87
Pt17	IGHV3-15	CARELGYYYYGMDVW	0.000636	99.32	IGHV3-74	CAREEGYYYYYMDVW	0.87
Pt17	IGHV3-64	CARELGYYYYGMDVW	0.000636	93.4	IGHV3-74	CAREEGYYYYYMDVW	0.87
Pt17	IGHV3-66	CARELGYYYYGMDVW	0.000636	94.04	IGHV3-74	CAREEGYYYYYMDVW	0.87
Pt17	IGHV3-11	CARELGYYYYGMDVW	0.20025	100	IGHV3-74	CAREEGYYYYYMDVW	0.87
Pt17	IGHV3-30	CARELGYYYYGMDVW	0.010807	98.61	IGHV3-74	CAREEGYYYYYMDVW	0.87
Pt17	IGHV3-30	CAREQGYYYYGMDVW	0.000636	99.65	IGHV3-74	CAREEGYYYYYMDVW	0.87
Pt17	IGHV3-33	CARGAGDGYYYGMDVW	0.003179	98.96	IGHV3-30-3	CARGYGGGYYYGMDVW	0.88
Pt17	IGHV3-7	CARGAGDGYYYGMDVW	0.003179	99.65	IGHV3-30-3	CARGYGGGYYYGMDVW	0.88
Pt17	IGHV3-48	CARGAGDGYYYGMDVW	0.001907	97.57	IGHV3-30-3	CARGYGGGYYYGMDVW	0.88
Pt17	IGHV3-53	CARGAGDGYYYGMDVW	0.001907	98.95	IGHV3-30-3	CARGYGGGYYYGMDVW	0.88
Pt17	IGHV3-74	CARGAGDGYYYGMDVW	0.001907	99.31	IGHV3-30-3	CARGYGGGYYYGMDVW	0.88
Pt17	IGHV3-30	CARGAGDGYYYGMDVW	0.065479	100	IGHV3-30-3	CARGYGGGYYYGMDVW	0.88
Pt17	IGHV3-11	CARGAGDGYYYGMDVW	0.001271	95.83	IGHV3-30-3	CARGYGGGYYYGMDVW	0.88
Pt17	IGHV3-13	CARGAGDGYYYGMDVW	0.001271	99.3	IGHV3-30-3	CARGYGGGYYYGMDVW	0.88
Pt17	IGHV3-66	CARGAGDGYYYGMDVW	0.001271	98.95	IGHV3-30-3	CARGYGGGYYYGMDVW	0.88
Pt17	IGHV3-21	CARGAGDGYYYGMDVW	0.000636	96.88	IGHV3-30-3	CARGYGGGYYYGMDVW	0.88
Pt17	IGHV3-64D	CARGAGDGYYYGMDVW	0.000636	99.31	IGHV3-30-3	CARGYGGGYYYGMDVW	0.88
Pt17	IGHV3-73	CARGAGDGYYYGMDVW	0.000636	100	IGHV3-30-3	CARGYGGGYYYGMDVW	0.88
Pt17	IGHV3-66	CARGAGDSYYYGMDVW	0.000636	98.25	IGHV3-30-3	CARGARGSYYYGMDVW	0.88
Pt17	IGHV3-7	CARGAGYYSGLDVW	0.000636	99.65	IGHV3-66	CARGAGYYYGMDVW	0.86
Pt17	IGHV3-74	CARGGQYYYGMDVW	0.000636	99.65	IGHV3-66	CARGAGYYYGMDVW	0.86
Pt17	IGHV3-33	CARGRSDIDAFDIW	0.000636	100	IGHV3-53	CARGRWEIDAFDIW	0.86
Pt17	IGHV3-74	CARGRSDIDAFDIW	0.000636	100	IGHV3-53	CARGRWEIDAFDIW	0.86
Pt17	IGHV3-11	CARIPTGNYYYGMDVW	0.000636	95.14	IGHV3-30	CARAFTGNYYYGMDVW	0.88

Pt17	IGHV3-48	CARIPTGNYYYGMDVW	0.000636	97.57	IGHV3-30	CARAFTGNYYYGMDVW	0.88
Pt17	IGHV3-48	CARISAGNYYYGMDVW	0.000636	94.44	IGHV3-30	CARGSAGNYYYGMDVW	0.94
Pt17	IGHV3-74	CARISTGNYYYGMDVW	0.008264	99.31	IGHV3-30	CARAFTGNYYYGMDVW	0.88
Pt17	IGHV3-33	CARISTGNYYYGMDVW	0.007629	97.22	IGHV3-30	CARAFTGNYYYGMDVW	0.88
Pt17	IGHV3-11	CARISTGNYYYGMDVW	0.44373	95.14	IGHV3-30	CARAFTGNYYYGMDVW	0.88
Pt17	IGHV3-66	CARISTGNYYYGMDVW	0.003814	97.54	IGHV3-30	CARAFTGNYYYGMDVW	0.88
Pt17	IGHV3-13	CARISTGNYYYGMDVW	0.003179	96.84	IGHV3-30	CARAFTGNYYYGMDVW	0.88
Pt17	IGHV3-64	CARISTGNYYYGMDVW	0.003179	95.49	IGHV3-30	CARAFTGNYYYGMDVW	0.88
Pt17	IGHV3-64D	CARISTGNYYYGMDVW	0.001907	98.61	IGHV3-30	CARAFTGNYYYGMDVW	0.88
Pt17	IGHV3-53	CARISTGNYYYGMDVW	0.001271	98.95	IGHV3-30	CARAFTGNYYYGMDVW	0.88
Pt17	IGHV3-48	CARISTGNYYYGMDVW	0.036872	95.83	IGHV3-30	CARAFTGNYYYGMDVW	0.88
Pt17	IGHV3-43	CARISTGNYYYGMDVW	0.000636	96.88	IGHV3-30	CARAFTGNYYYGMDVW	0.88
Pt17	IGHV3-7	CARISTGNYYYGMDVW	0.016529	96.53	IGHV3-30	CARAFTGNYYYGMDVW	0.88
Pt17	IGHV3-30	CARISTGNYYYGMDVW	0.015893	97.22	IGHV3-30	CARAFTGNYYYGMDVW	0.88
Pt17	IGHV3-21	CARISTGNYYYGMDVW	0.015257	94.1	IGHV3-30	CARAFTGNYYYGMDVW	0.88
Pt17	IGHV3-73	CARISTGNYYYGMDVW	0.000636	90.82	IGHV3-30	CARAFTGNYYYGMDVW	0.88
Pt17	IGHV3-NL1	CARISTGNYYYGMDVW	0.000636	96.84	IGHV3-30	CARAFTGNYYYGMDVW	0.88
Pt17	IGHV3-30	CARISTGSYYYGMDVW	0.000636	98.61	IGHV3-30	CARAYTGSYYYGMDVW	0.88
Pt17	IGHV3-11	CARTSTGNYYYGMDVW	0.000636	95.14	IGHV3-30	CARAFTGNYYYGMDVW	0.88
Pt17	IGHV3-33	CARTSTGNYYYGMDVW	0.000636	96.88	IGHV3-30	CARAFTGNYYYGMDVW	0.88
Pt17	IGHV3-21	CARVGCSSTSCYYYYYGMDVW	0.006357	100	IGHV3-9	CAKAGCSSTSCYYYYYYMDVW	0.86
Pt17	IGHV3-30	CARVGCSSTSCYYYYYGMDVW	0.000636	99.65	IGHV3-9	CAKAGCSSTSCYYYYYYMDVW	0.86
Pt17	IGHV3-48	CARVGCSSTSCYYYYYGMDVW	0.000636	95.83	IGHV3-9	CAKAGCSSTSCYYYYYYMDVW	0.86
Pt17	IGHV3-74	CARVGCSSTSCYYYYYGMDVW	0.000636	97.57	IGHV3-9	CAKAGCSSTSCYYYYYYMDVW	0.86
Pt18	IGHV3-30-3	CARDLTGDYYYGMDVW	0.053541	100	IGHV3-30	CARALTGGYYYGMDVW	0.88
Pt19	IGHV3-30-3	CARDGSSSWYFDLW	0.002116	100	IGHV3-7	CARVGSSSWYFDYW	0.86
Pt19	IGHV3-48	CARDKDYYGMDVW	0.002116	100	IGHV3-53	CARDGDYYGMDVW	0.92
Pt19	IGHV1-69	CARVDYW	0.008462	99.65	IGHV1-2	CARLDYW	0.86
Pt2	IGHV3-30	CAGALSPSYYYGMDVW	0.010434	100	IGHV3-30	CARALSGSYYYGMDVW	0.88
Pt2	IGHV3-23	CARDYGDYEVYFDYW	0.005217	100	IGHV3-53	CARGYGDYENYFDYW	0.87
Pt2	IGHV4-31	CARGFDYW	0.005217	95.88	IGHV4-4	CARGFDFW	0.88
Pt2	IGHV4-39	CARGFDYW	0.005217	100	IGHV4-4	CARGFDFW	0.88
Pt2	IGHV3-11	CARGLAATGAFDIW	0.005217	96.18	IGHV3-53	CARDLAAAGAFDIW	0.86
Pt2	IGHV3-30-3	CARGLAATGAFDIW	0.005217	100	IGHV3-53	CARDLAAAGAFDIW	0.86
Pt2	IGHV3-30	CARGLAATGAFDIW	0.43823	100	IGHV3-53	CARDLAAAGAFDIW	0.86
Pt2	IGHV3-48	CARGLAATGAFDIW	0.015651	99.31	IGHV3-53	CARDLAAAGAFDIW	0.86
Pt2	IGHV3-74	CARGLAATGAFDIW	0.015651	97.57	IGHV3-53	CARDLAAAGAFDIW	0.86
Pt2	IGHV3-33	CARGLAATGAFDIW	0.010434	99.65	IGHV3-53	CARDLAAAGAFDIW	0.86
Pt2	IGHV3-11	CARPYSGSYLGWFDPW	0.005217	96.88	IGHV3-30-3	CARPYSGSYNSWFDPW	0.88
Pt2	IGHV3-30-3	CARPYSGSYLGWFDPW	0.156511	97.22	IGHV3-30-3	CARPYSGSYNSWFDPW	0.88
Pt2	IGHV3-21	CARPYSGSYLGWFDPW	0.005217	98.61	IGHV3-30-3	CARPYSGSYNSWFDPW	0.88
Pt2	IGHV3-66	CARPYSGSYLGWFDPW	0.005217	96.84	IGHV3-30-3	CARPYSGSYNSWFDPW	0.88
Pt2	IGHV3-30	CARPYSGSYLGWFDPW	0.041736	97.22	IGHV3-30-3	CARPYSGSYNSWFDPW	0.88
Pt2	IGHV3-33	CARPYSGSYLGWFDPW	0.010434	98.96	IGHV3-30-3	CARPYSGSYNSWFDPW	0.88
Pt2	IGHV4-39	CARRTYGSGSYDYW	0.020868	99.66	IGHV4-4	CARRYYGSGSFDYW	0.86
Pt2	IGHV3-13	CASALSPSYYYGMDVW	0.005217	100	IGHV3-30	CARALSGSYYYGMDVW	0.88
Pt2	IGHV3-21	CASALSPSYYYGMDVW	0.005217	90.97	IGHV3-30	CARALSGSYYYGMDVW	0.88
Pt2	IGHV3-33	CASALSPSYYYGMDVW	0.005217	99.65	IGHV3-30	CARALSGSYYYGMDVW	0.88
Pt2	IGHV3-30	CASALSPSYYYGMDVW	0.422579	100	IGHV3-30	CARALSGSYYYGMDVW	0.88
Pt2	IGHV3-66	CASALSPSYYYGMDVW	0.005217	99.65	IGHV3-30	CARALSGSYYYGMDVW	0.88
Pt2	IGHV3-73	CASALSPSYYYGMDVW	0.005217	97.62	IGHV3-30	CARALSGSYYYGMDVW	0.88
Pt2	IGHV3-74	CASALSPSYYYGMDVW	0.005217	96.88	IGHV3-30	CARALSGSYYYGMDVW	0.88
Pt2	IGHV3-11	CASALSPSYYYGMDVW	0.020868	99.31	IGHV3-30	CARALSGSYYYGMDVW	0.88
Pt2	IGHV3-48	CASALSPSYYYGMDVW	0.015651	95.83	IGHV3-30	CARALSGSYYYGMDVW	0.88
Pt2	IGHV3-15	CASALSPSYYYGMDVW	0.010434	97.96	IGHV3-30	CARALSGSYYYGMDVW	0.88
Pt2	IGHV3-64	CASALSPSYYYGMDVW	0.010434	96.88	IGHV3-30	CARALSGSYYYGMDVW	0.88
Pt20	IGHV3-30-3	CARAYYYDSSGYYYYYYGMDVW	0.001607	100	IGHV3-13	CARANYFDSSGYGYYYYGMDVW	0.86
Pt20	IGHV3-7	CARAYYYDSSGYYYYYYGMDVW	0.001607	99.65	IGHV3-13	CARANYFDSSGYGYYYYGMDVW	0.86
Pt20	IGHV3-11	CARAYYYDSSGYYYYYYGMDVW	0.000804	99.65	IGHV3-13	CARANYFDSSGYGYYYYGMDVW	0.86
Pt20	IGHV3-13	CARAYYYDSSGYYYYYYGMDVW	0.000804	97.89	IGHV3-13	CARANYFDSSGYGYYYYGMDVW	0.86
Pt20	IGHV3-30	CARAYYYDSSGYYYYYYGMDVW	0.000804	100	IGHV3-13	CARANYFDSSGYGYYYYGMDVW	0.86
Pt20	IGHV3-33	CARAYYYDSSGYYYYYYGMDVW	0.000804	100	IGHV3-13	CARANYFDSSGYGYYYYGMDVW	0.86
Pt20	IGHV3-66	CARAYYYDSSGYYYYYYGMDVW	0.000804	98.6	IGHV3-13	CARANYFDSSGYGYYYYGMDVW	0.86
Pt20	IGHV3-30-3	CARDYAAAGTDYW	0.000804	99.31	IGHV3-66	CARDLAAAGTDYW	0.92
Pt20	IGHV4-34	CARGGYYDYW	0.000804	100	IGHV4-4	CARGWYYDYW	0.90
Pt20	IGHV3-66	CARKFLGNYYGMDVW	0.000804	99.65	IGHV3-30-3	CARGFGGNYYGMDVW	0.87
Pt20	IGHV3-21	CARLDYYDSSGSFDYW	0.001607	99.65	IGHV3-30	CAREDYHDSSGSFDYW	0.88
Pt20	IGHV3-30	CARLDYYDSSGSFDYW	0.001607	100	IGHV3-30	CAREDYHDSSGSFDYW	0.88
Pt20	IGHV3-66	CARLDYYDSSGSFDYW	0.001607	99.65	IGHV3-30	CAREDYHDSSGSFDYW	0.88
Pt20	IGHV3-74	CARLDYYDSSGSFDYW	0.001607	100	IGHV3-30	CAREDYHDSSGSFDYW	0.88
Pt20	IGHV3-11	CARLDYYDSSGSFDYW	0.000804	97.92	IGHV3-30	CAREDYHDSSGSFDYW	0.88
Pt20	IGHV3-13	CARLDYYDSSGSFDYW	0.000804	98.6	IGHV3-30	CAREDYHDSSGSFDYW	0.88
Pt20	IGHV3-15	CARLDYYDSSGSFDYW	0.000804	91.5	IGHV3-30	CAREDYHDSSGSFDYW	0.88
Pt20	IGHV3-48	CARLDYYDSSGSFDYW	0.03054	100	IGHV3-30	CAREDYHDSSGSFDYW	0.88
Pt20	IGHV3-7	CARLDYYDSSGSFDYW	0.000804	99.65	IGHV3-30	CAREDYHDSSGSFDYW	0.88
Pt22	IGHV4-61	CAKVSSYYYYMDVW	0.001707	97.59	IGHV4-30-2	CARVASYYYYMDVW	0.86
Pt22	IGHV3-30	CARAREPYYYYGMDVW	0.001707	99.65	IGHV3-30	CARAREGTYYYGMDVW	0.88

Pt22	IGHV3-30-3	CARDGGWYFDYW	0.001707	98.61	IGHV3-30-3	CARDGGGYFDYW	0.92
Pt22	IGHV3-53	CARDGGWYFDYW	0.001707	97.89	IGHV3-30-3	CARDGGGYFDYW	0.92
Pt22	IGHV3-72	CARDGGWYFDYW	0.001707	93.2	IGHV3-30-3	CARDGGGYFDYW	0.92
Pt22	IGHV3-73	CARDGGWYFDYW	0.001707	95.58	IGHV3-30-3	CARDGGGYFDYW	0.92
Pt22	IGHV3-74	CARDGGWYFDYW	0.001707	94.79	IGHV3-30-3	CARDGGGYFDYW	0.92
Pt22	IGHV3-30	CARDGGWYFDYW	0.020486	98.26	IGHV3-30-3	CARDGGGYFDYW	0.92
Pt22	IGHV3-33	CARDGGWYFDYW	0.010243	98.26	IGHV3-30-3	CARDGGGYFDYW	0.92
Pt22	IGHV3-11	CARDGGWYFDYW	0.008536	99.31	IGHV3-30-3	CARDGGGYFDYW	0.92
Pt22	IGHV3-13	CARDGGWYFDYW	0.005122	94.74	IGHV3-30-3	CARDGGGYFDYW	0.92
Pt22	IGHV3-48	CARDGGWYFDYW	0.380702	96.88	IGHV3-30-3	CARDGGGYFDYW	0.92
Pt22	IGHV3-33	CARDYGDNYYYGMDVW	0.001707	100	IGHV3-30-3	CARAYGGNYYYGMDVW	0.88
Pt22	IGHV3-33	CARDYGGDYYYGMDVW	0.001707	99.31	IGHV3-30-3	CARAYGGNYYYGMDVW	0.88
Pt23	IGHV3-11	CARGYGDSYFDYW	0.005547	95.83	IGHV3-53	CARGYGDLYFDYW	0.92
Pt23	IGHV3-30-3	CARGYGDSYFDYW	0.005547	98.26	IGHV3-53	CARGYGDLYFDYW	0.92
Pt24	IGHV3-7	CARDGGSSWIFDYW	0.008296	100	IGHV3-7	CARLGGSSWHFDYW	0.86
Pt24	IGHV3-48	CARDLNDLYGMDVW	0.008296	96.88	IGHV3-66	CARDLVVLYGMDVW	0.86
Pt24	IGHV3-53	CARDLNDLYGMDVW	0.008296	99.65	IGHV3-66	CARDLVVLYGMDVW	0.86
Pt24	IGHV3-30	CARDLNDLYGMDVW	0.04148	100	IGHV3-66	CARDLVVLYGMDVW	0.86
Pt24	IGHV3-11	CARDLNDLYGMDVW	0.008296	97.57	IGHV3-66	CARDLVVLYGMDVW	0.86
Pt24	IGHV3-21	CARGGDIVVVPAAMSYYYYGMDVW	0.016592	100	IGHV3-21	CARDLDIVVVPAARSYYYYGMDVW	0.88
Pt24	IGHV3-11	CARGGDIVVVPAAMSYYYYGMDVW	0.008296	100	IGHV3-21	CARDLDIVVVPAARSYYYYGMDVW	0.88
Pt25	IGHV6-1	CAREEQQLVYYYYYYGMDVW	0.001499	100	IGHV6-1	CAREEQQLVHDYYYYGMDVW	0.90
Pt25	IGHV3-43	CARETGAFDIW	0.001499	94.44	IGHV3-66	CARETYAFDIW	0.91
Pt25	IGHV3-30	CARGGITGTTFCDYW	0.001499	96.53	IGHV3-66	CARGGITGTTPIDYW	0.87
Pt25	IGHV3-30-3	CARGGITGTTFYDYW	0.005998	99.65	IGHV3-66	CARGGITGTTPIDYW	0.87
Pt25	IGHV3-7	CARGGITGTTFYDYW	0.005998	99.31	IGHV3-66	CARGGITGTTPIDYW	0.87
Pt25	IGHV3-30	CARGGITGTTFYDYW	0.089966	96.53	IGHV3-66	CARGGITGTTPIDYW	0.87
Pt25	IGHV3-33	CARGGITGTTFYDYW	0.002999	98.96	IGHV3-66	CARGGITGTTPIDYW	0.87
Pt25	IGHV3-48	CARGGITGTTFYDYW	0.002999	97.92	IGHV3-66	CARGGITGTTPIDYW	0.87
Pt25	IGHV3-13	CARGGITGTTFYDYW	0.001499	96.49	IGHV3-66	CARGGITGTTPIDYW	0.87
Pt25	IGHV3-21	CARGGITGTTFYDYW	0.001499	97.22	IGHV3-66	CARGGITGTTPIDYW	0.87
Pt25	IGHV3-64	CARGGITGTTFYDYW	0.001499	94.44	IGHV3-66	CARGGITGTTPIDYW	0.87
Pt25	IGHV3-66	CARGGITGTTFYDYW	0.001499	99.65	IGHV3-66	CARGGITGTTPIDYW	0.87
Pt25	IGHV3-NL1	CARGGITGTTFYDYW	0.001499	97.89	IGHV3-66	CARGGITGTTPIDYW	0.87
Pt25	IGHV3-30	CARGGITGTTSYDYW	0.001499	97.92	IGHV3-66	CARGGITGTTPIDYW	0.87
Pt25	IGHV3-30	CARGGYYYYGMDVW	0.010496	99.31	IGHV3-66	CARGAGYYYGMDVW	0.86
Pt25	IGHV3-30-3	CARGGYYYYGMDVW	0.004498	99.31	IGHV3-66	CARGAGYYYGMDVW	0.86
Pt25	IGHV3-33	CARGGYYYYGMDVW	0.004498	100	IGHV3-66	CARGAGYYYGMDVW	0.86
Pt25	IGHV3-53	CARGGYYYYGMDVW	0.002999	98.95	IGHV3-66	CARGAGYYYGMDVW	0.86
Pt25	IGHV3-66	CARGGYYYYGMDVW	0.002999	99.3	IGHV3-66	CARGAGYYYGMDVW	0.86
Pt25	IGHV3-7	CARGGYYYYGMDVW	0.002999	98.96	IGHV3-66	CARGAGYYYGMDVW	0.86
Pt25	IGHV3-48	CARGGYYYYGMDVW	0.046482	100	IGHV3-66	CARGAGYYYGMDVW	0.86
Pt25	IGHV3-11	CARGGYYYYGMDVW	0.001499	98.26	IGHV3-66	CARGAGYYYGMDVW	0.86
Pt25	IGHV3-20	CARGGYYYYGMDVW	0.001499	97.92	IGHV3-66	CARGAGYYYGMDVW	0.86
Pt25	IGHV3-21	CARGGYYYYGMDVW	0.001499	99.31	IGHV3-66	CARGAGYYYGMDVW	0.86
Pt25	IGHV3-74	CARGGYYYYGMDVW	0.001499	97.57	IGHV3-66	CARGAGYYYGMDVW	0.86
Pt26	IGHV3-30-3	CAKDRDYYDSSGYCDYW	0.003288	96.53	IGHV3-9	CAKDYDYYDSSGYFDYW	0.88
Pt26	IGHV3-53	CARESGYYGMDVW	0.006576	100	IGHV3-53	CARELGYYGMDVW	0.92
Pt26	IGHV3-66	CARESGYYGMDVW	0.006576	97.19	IGHV3-53	CARELGYYGMDVW	0.92
Pt26	IGHV3-15	CARESGYYGMDVW	0.003288	94.9	IGHV3-53	CARELGYYGMDVW	0.92
Pt26	IGHV3-33	CARESGYYGMDVW	0.003288	98.26	IGHV3-53	CARELGYYGMDVW	0.92
Pt26	IGHV3-49	CARESGYYGMDVW	0.003288	92.86	IGHV3-53	CARELGYYGMDVW	0.92
Pt26	IGHV3-64	CARESGYYGMDVW	0.003288	99.31	IGHV3-53	CARELGYYGMDVW	0.92
Pt26	IGHV3-74	CARESGYYGMDVW	0.003288	97.22	IGHV3-53	CARELGYYGMDVW	0.92
Pt26	IGHV3-11	CARESGYYGMDVW	0.361699	100	IGHV3-53	CARELGYYGMDVW	0.92
Pt26	IGHV3-30	CARESGYYGMDVW	0.013153	98.96	IGHV3-53	CARELGYYGMDVW	0.92
Pt26	IGHV3-7	CARESGYYGMDVW	0.013153	96.53	IGHV3-53	CARELGYYGMDVW	0.92
Pt26	IGHV3-30-3	CARESGYYGMDVW	0.009865	100	IGHV3-53	CARELGYYGMDVW	0.92
Pt26	IGHV3-48	CARESGYYGMDVW	0.009865	97.22	IGHV3-53	CARELGYYGMDVW	0.92
Pt27	IGHV3-33	CARDYYYGMDVW	0.007293	100	IGHV3-66	CAREYYYGMDVW	0.92
Pt27	IGHV3-7	CARDYYYYGMDVW	0.007293	97.92	IGHV3-66	CARDLYYYGMDVW	0.92
Pt27	IGHV3-30	CARDYYYYGMDVW	0.04376	99.65	IGHV3-66	CARDLYYYGMDVW	0.92
Pt27	IGHV3-30-3	CARDYYYYGMDVW	0.02188	97.92	IGHV3-66	CARDLYYYGMDVW	0.92
Pt27	IGHV3-48	CARDYYYYGMDVW	0.014587	98.26	IGHV3-66	CARDLYYYGMDVW	0.92
Pt27	IGHV3-20	CARDYYYYGMDVW	0.007293	98.26	IGHV3-66	CARDLYYYGMDVW	0.92
Pt27	IGHV3-21	CARDYYYYGMDVW	0.007293	98.61	IGHV3-66	CARDLYYYGMDVW	0.92
Pt27	IGHV3-33	CARDYYYYGMDVW	1.662898	100	IGHV3-66	CARDLYYYGMDVW	0.92
Pt27	IGHV3-53	CARDYYYYGMDVW	0.007293	95.09	IGHV3-66	CARDLYYYGMDVW	0.92
Pt27	IGHV3-30-3	CARENYGMDVW	0.007293	99.65	IGHV3-53	CAREAYGMDVW	0.91
Pt27	IGHV3-48	CARGDYYYGMDVW	0.014587	99.65	IGHV3-66	CARGAGYYYGMDVW	0.86
Pt27	IGHV3-21	CARGMGYYYGMDVW	0.014587	98.26	IGHV3-66	CARGAGYYYGMDVW	0.93
Pt29	IGHV3-48	CARDRGGTGYYYYGMDVW	0.001626	95.49	IGHV3-48	CARDRGGYGPYYYGMDVW	0.89
Pt29	IGHV3-11	CARDRGGTGYYYYGMDVW	0.026015	99.65	IGHV3-48	CARDRGGYGPYYYGMDVW	0.89
Pt29	IGHV3-33	CAREYGANYYGMDVW	0.001626	95.83	IGHV3-66	CARGEGANYYGMDVW	0.87
Pt29	IGHV3-33	CARGYGAYYYGMDVW	0.003252	95.83	IGHV3-66	CARGEGANYYGMDVW	0.87
Pt3	IGHV4-61	CARAESYYYYMDVW	0.002009	98.97	IGHV4-30-2	CARVASYYYYMDVW	0.86
Pt3	IGHV4-59	CARAESYYYYMDVW	0.036154	100	IGHV4-30-2	CARVASYYYYMDVW	0.86

Pt3	IGHV4-59	CARAGSYYYYMDVW	0.002009	97.19	IGHV4-34	CARVGGYYYYYMDVW	0.87
Pt3	IGHV4-61	CARDFDPW	0.168719	100	IGHV4-59	CARGFDPW	0.88
Pt3	IGHV4-34	CARDFDPW	0.002009	99.65	IGHV4-59	CARGFDPW	0.88
Pt3	IGHV4-4	CARDFDPW	0.002009	99.3	IGHV4-59	CARGFDPW	0.88
Pt3	IGHV4-59	CARDFDPW	0.002009	100	IGHV4-59	CARGFDPW	0.88
Pt3	IGHV4-39	CARDFDPW	0.010043	97.94	IGHV4-59	CARGFDPW	0.88
Pt3	IGHV3-53	CARDGSDAFDIW	0.004017	99.3	IGHV3-53	CARDISDAFDIW	0.92
Pt3	IGHV3-66	CARDGSDAFDIW	0.004017	99.65	IGHV3-53	CARDISDAFDIW	0.92
Pt3	IGHV3-33	CARDGSDAFDIW	0.158676	100	IGHV3-53	CARDISDAFDIW	0.92
Pt3	IGHV3-21	CARDGSDAFDIW	0.002009	99.31	IGHV3-53	CARDISDAFDIW	0.92
Pt3	IGHV3-64	CARDGSDAFDIW	0.002009	92.28	IGHV3-53	CARDISDAFDIW	0.92
Pt3	IGHV3-7	CARDGSDAFDIW	0.002009	98.61	IGHV3-53	CARDISDAFDIW	0.92
Pt3	IGHV3-30	CARDGSDAFDIW	0.008034	99.65	IGHV3-53	CARDISDAFDIW	0.92
Pt3	IGHV3-33	CARGNYYYYGMDVW	0.004017	100	IGHV3-66	CARGAGYYYGMDVW	0.86
Pt3	IGHV3-66	CARGSEYYYGMDVW	0.002009	99.65	IGHV3-66	CARGAGYYYGMDVW	0.86
Pt30	IGHV4-34	CARAYCGGDCYPDYW	0.005444	100	IGHV4-30-2	CARAYCGGDCPFDYW	0.87
Pt30	IGHV4-30-4	CARAYCGGDCYPDYW	0.002722	100	IGHV4-30-2	CARAYCGGDCPFDYW	0.87
Pt30	IGHV4-30-2	CARAYCGGDCYPDYW	0.019055	100	IGHV4-30-2	CARAYCGGDCPFDYW	0.87
Pt30	IGHV3-30-3	CARDLNIYGMDVW	0.002722	100	IGHV3-53	CARDLHIYGMDVW	0.92
Pt30	IGHV3-30-3	CARDSSGYYYFDYW	0.016333	99.65	IGHV3-30-3	CARDHSGYDYFDYW	0.86
Pt30	IGHV3-30	CARDSSGYYYFDYW	0.002722	99.31	IGHV3-30-3	CARDHSGYDYFDYW	0.86
Pt30	IGHV3-64D	CARDSSGYYYFDYW	0.002722	100	IGHV3-30-3	CARDHSGYDYFDYW	0.86
Pt30	IGHV3-74	CARGAFYFDYW	0.013611	100	IGHV3-72	CARGPFYFDYW	0.91
Pt30	IGHV3-11	CARGAFYFDYW	0.002722	98.96	IGHV3-72	CARGPFYFDYW	0.91
Pt30	IGHV3-48	CARGAFYFDYW	0.002722	99.31	IGHV3-72	CARGPFYFDYW	0.91
Pt30	IGHV3-33	CARGPYCSSTSCFYYYGMDAW	0.002722	99.31	IGHV3-72	CARGPYCSSTSCYYYYGMDVW	0.90
Pt30	IGHV3-7	CARGPYCSSTSCFYYYGMDVW	0.065333	100	IGHV3-72	CARGPYCSSTSCYYYYGMDVW	0.95
Pt30	IGHV3-30-3	CARGPYCSSTSCFYYYGMDVW	0.005444	95.83	IGHV3-72	CARGPYCSSTSCYYYYGMDVW	0.95
Pt30	IGHV3-11	CARGPYCSSTSCFYYYGMDVW	0.002722	98.96	IGHV3-72	CARGPYCSSTSCYYYYGMDVW	0.95
Pt30	IGHV3-13	CARGPYCSSTSCFYYYGMDVW	0.002722	97.19	IGHV3-72	CARGPYCSSTSCYYYYGMDVW	0.95
Pt30	IGHV3-21	CARGPYCSSTSCFYYYGMDVW	0.002722	98.26	IGHV3-72	CARGPYCSSTSCYYYYGMDVW	0.95
Pt30	IGHV3-30	CARGPYCSSTSCFYYYGMDVW	0.002722	98.96	IGHV3-72	CARGPYCSSTSCYYYYGMDVW	0.95
Pt30	IGHV3-53	CARGPYCSSTSCFYYYGMDVW	0.002722	97.89	IGHV3-72	CARGPYCSSTSCYYYYGMDVW	0.95
Pt30	IGHV1-46	CARGSGSSVYNWFDPW	0.005444	100	IGHV1-69	CARGGGSSGYNWFDPW	0.88
Pt30	IGHV3-30	CARSLGGSYYYGMDVW	0.008167	99.31	IGHV3-30-3	CAKSLGGSYYYGMDVW	0.94
Pt30	IGHV3-30-3	CARSLGGSYYYGMDVW	0.059888	97.92	IGHV3-30-3	CAKSLGGSYYYGMDVW	0.94
Pt30	IGHV3-13	CARSLGGSYYYGMDVW	0.002722	98.6	IGHV3-30-3	CAKSLGGSYYYGMDVW	0.94
Pt30	IGHV3-21	CARSLGGSYYYGMDVW	0.002722	96.88	IGHV3-30-3	CAKSLGGSYYYGMDVW	0.94
Pt30	IGHV3-33	CARSLGGSYYYGMDVW	0.002722	98.96	IGHV3-30-3	CAKSLGGSYYYGMDVW	0.94
Pt30	IGHV3-48	CARSLGGSYYYGMDVW	0.002722	99.31	IGHV3-30-3	CAKSLGGSYYYGMDVW	0.94
Pt30	IGHV3-66	CARSLGGSYYYGMDVW	0.002722	98.95	IGHV3-30-3	CAKSLGGSYYYGMDVW	0.94
Pt30	IGHV3-7	CARSLGGSYYYGMDVW	0.002722	98.96	IGHV3-30-3	CAKSLGGSYYYGMDVW	0.94
Pt30	IGHV3-30-3	CARSLGGSYYYGMNVW	0.002722	97.92	IGHV3-30-3	CAKSLGGSYYYGMDVW	0.88
Pt30	IGHV2-70	CARTMSGYYYYGMDVW	0.013611	100	IGHV2-70	CARTHSDYYYYGMDVW	0.88
Pt30	IGHV3-66	CARWLQYYGMDVW	0.008167	97.89	IGHV3-53	CARDLQYYGMDVW	0.92
Pt30	IGHV3-11	CARWLQYYGMDVW	0.005444	99.31	IGHV3-53	CARDLQYYGMDVW	0.92
Pt30	IGHV3-13	CARWLQYYGMDVW	0.005444	99.65	IGHV3-53	CARDLQYYGMDVW	0.92
Pt30	IGHV3-30	CARWLQYYGMDVW	0.005444	99.31	IGHV3-53	CARDLQYYGMDVW	0.92
Pt30	IGHV3-30-3	CARWLQYYGMDVW	0.005444	98.61	IGHV3-53	CARDLQYYGMDVW	0.92
Pt30	IGHV3-NL1	CARWLQYYGMDVW	0.005444	97.19	IGHV3-53	CARDLQYYGMDVW	0.92
Pt30	IGHV3-15	CARWLQYYGMDVW	0.002722	96.6	IGHV3-53	CARDLQYYGMDVW	0.92
Pt30	IGHV3-21	CARWLQYYGMDVW	0.002722	97.22	IGHV3-53	CARDLQYYGMDVW	0.92
Pt30	IGHV3-23	CARWLQYYGMDVW	0.002722	93.33	IGHV3-53	CARDLQYYGMDVW	0.92
Pt30	IGHV3-64	CARWLQYYGMDVW	0.002722	97.57	IGHV3-53	CARDLQYYGMDVW	0.92
Pt30	IGHV3-7	CARWLQYYGMDVW	0.002722	98.26	IGHV3-53	CARDLQYYGMDVW	0.92
Pt30	IGHV3-74	CARWLQYYGMDVW	0.002722	98.61	IGHV3-53	CARDLQYYGMDVW	0.92
Pt30	IGHV3-53	CARWLQYYGMDVW	0.106166	100	IGHV3-53	CARDLQYYGMDVW	0.92
Pt30	IGHV3-33	CARWLQYYGMDVW	0.021778	99.31	IGHV3-53	CARDLQYYGMDVW	0.92
Pt31	IGHV3-33	CARDFRITMVQGVSYYYYGMDVW	0.005916	98.96	IGHV3-53	CARDRRITMVRGVIYYYYGMDVW	0.87
Pt31	IGHV3-48	CARDFRITMVQGVSYYYYGMDVW	0.017749	100	IGHV3-53	CARDRRITMVRGVIYYYYGMDVW	0.87
Pt34	IGHV3-49	CAREGDGYNYKFDYW	0.00186	94.22	IGHV3-23	CAREGDGYNFYFDYW	0.87
Pt34	IGHV1-18	CARGLFDYW	0.024182	100	IGHV1-18	CARGKFDYW	0.89
Pt35	IGHV3-21	CAREEIPYYYYMDVW	0.001681	98.61	IGHV3-74	CAREEGYYYYYMDVW	0.87
Pt35	IGHV3-33	CAREEIPYYYYMDVW	0.001121	97.92	IGHV3-74	CAREEGYYYYYMDVW	0.87
Pt35	IGHV3-48	CAREEIPYYYYMDVW	0.001121	97.92	IGHV3-74	CAREEGYYYYYMDVW	0.87
Pt35	IGHV3-13	CAREEIPYYYYMDVW	0.00056	92.98	IGHV3-74	CAREEGYYYYYMDVW	0.87
Pt35	IGHV3-49	CAREEIPYYYYMDVW	0.00056	94.22	IGHV3-74	CAREEGYYYYYMDVW	0.87
Pt35	IGHV3-7	CAREEIPYYYYMDVW	0.00056	99.65	IGHV3-74	CAREEGYYYYYMDVW	0.87
Pt35	IGHV3-74	CAREEIPYYYYMDVW	0.00056	92.36	IGHV3-74	CAREEGYYYYYMDVW	0.87
Pt35	IGHV3-11	CAREEIPYYYYMDVW	0.012331	100	IGHV3-74	CAREEGYYYYYMDVW	0.87
Pt35	IGHV4-39	CARGGLYGDYYDYW	0.00056	100	IGHV4-59	CARGGDYGDYFDYW	0.86
Pt35	IGHV3-53	CARGKQQLVPYYYYYYMDVW	0.001121	98.25	IGHV3-13	CARGKQQLVRAYYYYYMDVW	0.90
Pt35	IGHV3-7	CARGKQQLVPYYYYYYMDVW	0.00056	98.96	IGHV3-13	CARGKQQLVRAYYYYYMDVW	0.90
Pt36	IGHV3-NL1	CARDRGGYFDYW	0.004576	97.89	IGHV3-30-3	CARDGGGYFDYW	0.92
Pt36	IGHV3-53	CARDRGGYFDYW	0.139563	100	IGHV3-30-3	CARDGGGYFDYW	0.92
Pt36	IGHV3-13	CARDRGGYFDYW	0.002288	97.89	IGHV3-30-3	CARDGGGYFDYW	0.92
Pt36	IGHV3-23	CARDRGGYFDYW	0.002288	92.01	IGHV3-30-3	CARDGGGYFDYW	0.92

Pt36	IGHV3-49	CARDRGGYFDYW	0.002288	98.64	IGHV3-30-3	CARDGGGYFDYW	0.92
Pt36	IGHV3-30	CARDRGGYFDYW	0.027455	98.96	IGHV3-30-3	CARDGGGYFDYW	0.92
Pt36	IGHV3-66	CARDRGGYFDYW	0.020591	97.54	IGHV3-30-3	CARDGGGYFDYW	0.92
Pt36	IGHV3-33	CARDRGGYFDYW	0.009152	98.96	IGHV3-30-3	CARDGGGYFDYW	0.92
Pt36	IGHV3-11	CARDRGGYFDYW	0.006864	98.96	IGHV3-30-3	CARDGGGYFDYW	0.92
Pt36	IGHV3-74	CARDRGGYFDYW	0.006864	97.57	IGHV3-30-3	CARDGGGYFDYW	0.92
Pt36	IGHV3-13	CARRGYGGYYHGMDVW	0.002288	98.6	IGHV3-30-3	CARGQYGGYYHGMDVW	0.88
Pt36	IGHV3-30	CARVRYGGYVEYFDYW	0.002288	95.49	IGHV3-13	CARVKYGGYVGYFDYW	0.88
Pt37	IGHV3-48	CARDRGWNYLFDYW	0.004142	100	IGHV3-48	CARDRGWNYGLDYW	0.86
Pt37	IGHV3-21	CARDRGWNYLFDYW	0.001381	99.31	IGHV3-48	CARDRGWNYGLDYW	0.86
Pt37	IGHV3-33	CARDRGWNYLFDYW	0.001381	99.65	IGHV3-48	CARDRGWNYGLDYW	0.86
Pt37	IGHV3-49	CARDRGWNYLFDYW	0.001381	98.3	IGHV3-48	CARDRGWNYGLDYW	0.86
Pt37	IGHV3-53	CARDSYYDSSGSFDYW	0.008284	98.25	IGHV3-30	CAREDYYDSSGSFDYW	0.88
Pt37	IGHV3-33	CARDSYYDSSGSFDYW	0.006903	99.65	IGHV3-30	CAREDYYDSSGSFDYW	0.88
Pt37	IGHV3-21	CARDSYYDSSGSFDYW	0.005522	99.31	IGHV3-30	CAREDYYDSSGSFDYW	0.88
Pt37	IGHV3-11	CARDSYYDSSGSFDYW	0.146346	100	IGHV3-30	CAREDYYDSSGSFDYW	0.88
Pt37	IGHV3-30	CARDSYYDSSGSFDYW	0.004142	100	IGHV3-30	CAREDYYDSSGSFDYW	0.88
Pt37	IGHV3-30-3	CARDSYYDSSGSFDYW	0.004142	99.65	IGHV3-30	CAREDYYDSSGSFDYW	0.88
Pt37	IGHV3-7	CARDSYYDSSGSFDYW	0.002761	96.53	IGHV3-30	CAREDYYDSSGSFDYW	0.88
Pt37	IGHV3-13	CARDSYYDSSGSFDYW	0.001381	97.54	IGHV3-30	CAREDYYDSSGSFDYW	0.88
Pt37	IGHV3-43D	CARDSYYDSSGSFDYW	0.001381	98.61	IGHV3-30	CAREDYYDSSGSFDYW	0.88
Pt37	IGHV3-66	CARDSYYDSSGSFDYW	0.001381	97.54	IGHV3-30	CAREDYYDSSGSFDYW	0.88
Pt37	IGHV3-48	CARDSYYDSSGSFDYW	0.013806	98.96	IGHV3-30	CAREDYYDSSGSFDYW	0.88
Pt37	IGHV1-2	CAREFCSGGSCYSGNWFDPW	0.019329	100	IGHV1-69	CAREFCSGGSCSYNNWFDPW	0.85
Pt37	IGHV3-33	CARGSGSTSYYYYGMDVW	0.001381	100	IGHV3-48	CARGVGPTSYYYYGMDVW	0.89
Pt37	IGHV3-30-3	CARVSGSTSYYYYGMDVW	0.004142	99.65	IGHV3-48	CARVSGYRSYYYYGMDVW	0.89
Pt38	IGHV4-34	CARDPHYDFWSGYPYYYYYGMDVW	0.005746	100	IGHV4-30-4	CARDHHYDFWSGYSSYYYYGMDVW	0.88
Pt38	IGHV4-39	CARDPHYDFWSGYPYYYYYGMDVW	0.002873	97.59	IGHV4-30-4	CARDHHYDFWSGYSSYYYYGMDVW	0.88
Pt39	IGHV3-30	CARDWGYYYYYMDVW	0.001823	99.31	IGHV3-74	CAREEGYYYYYMDVW	0.87
Pt39	IGHV3-30	CARIWGYYYYYMDVW	0.001823	93.4	IGHV3-74	CAREEGYYYYYMDVW	0.87
Pt39	IGHV4-39	CARVWGYYYYMDVW	0.001823	79.38	IGHV4-30-2	CARVASYYYYMDVW	0.86
Pt39	IGHV3-66	CARVWGYYYYYMDVW	0.005469	99.3	IGHV3-74	CAREEGYYYYYMDVW	0.87
Pt39	IGHV3-30	CARVWGYYYYYMDVW	0.149485	99.31	IGHV3-74	CAREEGYYYYYMDVW	0.87
Pt39	IGHV3-43D	CARVWGYYYYYMDVW	0.003646	98.96	IGHV3-74	CAREEGYYYYYMDVW	0.87
Pt39	IGHV4-30-4	CARVWGYYYYYMDVW	0.003646	99.66	IGHV4-34	CARVGGYYYYYMDVW	0.93
Pt39	IGHV4-34	CARVWGYYYYYMDVW	0.003646	100	IGHV4-34	CARVGGYYYYYMDVW	0.93
Pt39	IGHV3-11	CARVWGYYYYYMDVW	0.001823	97.22	IGHV3-74	CAREEGYYYYYMDVW	0.87
Pt39	IGHV3-15	CARVWGYYYYYMDVW	0.001823	98.3	IGHV3-74	CAREEGYYYYYMDVW	0.87
Pt39	IGHV3-33	CARVWGYYYYYMDVW	0.001823	100	IGHV3-74	CAREEGYYYYYMDVW	0.87
Pt39	IGHV3-7	CARVWGYYYYYMDVW	0.001823	94.44	IGHV3-74	CAREEGYYYYYMDVW	0.87
Pt39	IGHV3-73	CARVWGYYYYYMDVW	0.001823	98.98	IGHV3-74	CAREEGYYYYYMDVW	0.87
Pt39	IGHV3-30-3	CARVWGYYYYYMDVW	0.470331	100	IGHV3-74	CAREEGYYYYYMDVW	0.87
Pt39	IGHV3-74	CARVWGYYYYYMDVW	0.001823	99.31	IGHV3-74	CAREEGYYYYYMDVW	0.87
Pt39	IGHV3-NL1	CARVWGYYYYYMDVW	0.001823	96.84	IGHV3-74	CAREEGYYYYYMDVW	0.87
Pt39	IGHV4-31	CARVWGYYYYYMDVW	0.001823	90.72	IGHV4-34	CARVGGYYYYYMDVW	0.93
Pt39	IGHV4-39	CARVWGYYYYYMDVW	0.001823	98.95	IGHV4-34	CARVGGYYYYYMDVW	0.93
Pt39	IGHV4-59	CARVWGYYYYYMDVW	0.001823	100	IGHV4-34	CARVGGYYYYYMDVW	0.93
Pt39	IGHV3-48	CARVWGYYYYYMDVW	0.010938	99.31	IGHV3-74	CAREEGYYYYYMDVW	0.87
Pt39	IGHV4-4	CARVWGYYYYYMDVW	0.007292	98.96	IGHV4-34	CARVGGYYYYYMDVW	0.93
Pt4	IGHV3-30	CAKAAGGYYYYGMDVW	0.004103	97.22	IGHV3-30	CAKARGGSYYYGMDVW	0.88
Pt4	IGHV3-23	CAKAAGGYYYYGMDVW	0.012309	100	IGHV3-30	CAKARGGSYYYGMDVW	0.88
Pt4	IGHV3-33	CARGGIILADAFDIW	0.004103	99.65	IGHV3-53	CARGGIIAWDAFDIW	0.87
Pt4	IGHV3-30-3	CARGGIILADAFDIW	0.020515	100	IGHV3-53	CARGGIIAWDAFDIW	0.87
Pt4	IGHV3-30-3	CARNYYDSSDAFDIW	0.102577	98.96	IGHV3-30	CARNYYDSSDAFDIW	1.00
Pt4	IGHV3-33	CARNYYDSSDAFDIW	0.004103	99.31	IGHV3-30	CARNYYDSSDAFDIW	1.00
Pt4	IGHV3-66	CARNYYDSSDAFDIW	0.004103	95.44	IGHV3-30	CARNYYDSSDAFDIW	1.00
Pt4	IGHV3-30	CARNYYDSSDAFDIW	0.016412	99.65	IGHV3-30	CARNYYDSSDAFDIW	1.00
Pt4	IGHV3-30	CARSYYDSSDAFDIW	0.004103	98.61	IGHV3-30	CARNYYDSSDAFDIW	0.93
Pt40	IGHV3-74	CARDKKDYYGMDVW	0.001078	100	IGHV3-53	CARDVGDYYGMDVW	0.86
Pt40	IGHV3-21	CARVRSDAFDIW	0.001078	99.65	IGHV3-53	CARVVSDAFDIW	0.92
Pt41	IGHV3-30	CARGLSSSGYFDYW	0.006132	95.83	IGHV3-53	CARDLSSSGGFDYW	0.86
Pt41	IGHV3-7	CARGLSSSGYFDYW	0.003066	96.18	IGHV3-53	CARDLSSSGGFDYW	0.86
Pt41	IGHV3-11	CARGLSSSGYFDYW	0.001533	94.1	IGHV3-53	CARDLSSSGGFDYW	0.86
Pt41	IGHV3-33	CARGLSSSGYFDYW	0.001533	99.65	IGHV3-53	CARDLSSSGGFDYW	0.86
Pt41	IGHV3-48	CARGLSSSGYFDYW	0.001533	97.22	IGHV3-53	CARDLSSSGGFDYW	0.86
Pt41	IGHV3-64	CARGLSSSGYFDYW	0.036793	100	IGHV3-53	CARDLSSSGGFDYW	0.86
Pt41	IGHV3-74	CARGLSSSGYFDYW	0.001533	97.57	IGHV3-53	CARDLSSSGGFDYW	0.86
Pt41	IGHV4-59	CARGQLAYDAFDIW	0.421592	100	IGHV4-59	CARGQLRGDAFDIW	0.86
Pt41	IGHV4-61	CARGQLAYDAFDIW	0.082785	98.63	IGHV4-59	CARGQLRGDAFDIW	0.86
Pt41	IGHV4-34	CARGQLAYDAFDIW	0.050591	99.65	IGHV4-59	CARGQLRGDAFDIW	0.86
Pt41	IGHV4-39	CARGQLAYDAFDIW	0.03986	96.56	IGHV4-59	CARGQLRGDAFDIW	0.86
Pt41	IGHV4-31	CARGQLAYDAFDIW	0.021463	99.31	IGHV4-59	CARGQLRGDAFDIW	0.86
Pt41	IGHV4-4	CARGQLAYDAFDIW	0.013798	99.65	IGHV4-59	CARGQLRGDAFDIW	0.86
Pt41	IGHV4-30-4	CARGQLAYDAFDIW	0.007665	98.28	IGHV4-59	CARGQLRGDAFDIW	0.86
Pt41	IGHV4-59	CARGQLDYDAFDIW	0.001533	95.44	IGHV4-59	CARGQLRGDAFDIW	0.86
Pt41	IGHV3-74	CARGSGWYFYFDYW	0.003066	99.65	IGHV3-48	CARGSGWYVGFDYW	0.86
Pt41	IGHV3-11	CARGSGWYFYFDYW	0.001533	98.26	IGHV3-48	CARGSGWYVGFDYW	0.86

Pt41	IGHV3-30-3	CARGSGWYFYFDYW	0.001533	97.92	IGHV3-48	CARGSGWYVGFDYW	0.86
Pt41	IGHV3-33	CARGSGWYFYFDYW	0.001533	100	IGHV3-48	CARGSGWYVGFDYW	0.86
Pt41	IGHV3-7	CARGSGWYFYFDYW	0.001533	95.14	IGHV3-48	CARGSGWYVGFDYW	0.86
Pt41	IGHV3-66	CARIGPYYYGMDVW	0.001533	95.44	IGHV3-53	CARGGPYYYAMDVW	0.86
Pt41	IGHV4-28	CARVGSSYYYYMDVW	0.001533	99.31	IGHV4-34	CARVGGYYYYYMDVW	0.87
Pt41	IGHV4-30-4	CARVGSSYYYYMDVW	0.001533	98.97	IGHV4-34	CARVGGYYYYYMDVW	0.87
Pt41	IGHV4-34	CARVGSSYYYYMDVW	0.018397	99.65	IGHV4-34	CARVGGYYYYYMDVW	0.87
Pt41	IGHV4-4	CARVGSSYYYYMDVW	0.018397	99.65	IGHV4-34	CARVGGYYYYYMDVW	0.87
Pt41	IGHV4-59	CARVGSSYYYYMDVW	0.018397	99.3	IGHV4-34	CARVGGYYYYYMDVW	0.87
Pt41	IGHV4-61	CARVGSSYYYYMDVW	0.251422	99.66	IGHV4-34	CARVGGYYYYYMDVW	0.87
Pt41	IGHV4-39	CARVGSSYYYYMDVW	0.012264	100	IGHV4-34	CARVGGYYYYYMDVW	0.87
Pt41	IGHV4-31	CARVGSSYYYYMDVW	0.010731	98.28	IGHV4-34	CARVGGYYYYYMDVW	0.87
Pt42	IGHV3-30	CAKSGGYGSSWDFDYW	0.001857	99.31	IGHV3-30	CAKSAGYTSSWDFDYW	0.88
Pt42	IGHV3-20	CAKSGGYSSSWDFDYW	0.001857	90.97	IGHV3-30	CAKSAGYTSSWDFDYW	0.88
Pt42	IGHV3-43	CAKSGGYSSSWDFDYW	0.001857	94.79	IGHV3-30	CAKSAGYTSSWDFDYW	0.88
Pt42	IGHV3-48	CAKSGGYSSSWDFDYW	0.001857	93.06	IGHV3-30	CAKSAGYTSSWDFDYW	0.88
Pt42	IGHV3-NL1	CAKSGGYSSSWDFDYW	0.001857	91.32	IGHV3-30	CAKSAGYTSSWDFDYW	0.88
Pt42	IGHV3-30-3	CAKSGGYSSSWDFDYW	0.024142	99.65	IGHV3-30	CAKSAGYTSSWDFDYW	0.88
Pt42	IGHV3-33	CAKSGGYSSSWDFDYW	0.011142	98.96	IGHV3-30	CAKSAGYTSSWDFDYW	0.88
Pt42	IGHV3-7	CAKSGGYSSSWDFDYW	0.007428	96.53	IGHV3-30	CAKSAGYTSSWDFDYW	0.88
Pt42	IGHV3-30	CAKSGGYSSSWDFDYW	0.427128	99.31	IGHV3-30	CAKSAGYTSSWDFDYW	0.88
Pt42	IGHV3-13	CAKSGGYSSSWDFDYW	0.005571	98.6	IGHV3-30	CAKSAGYTSSWDFDYW	0.88
Pt42	IGHV3-21	CAKSGGYSSSWDFDYW	0.005571	97.35	IGHV3-30	CAKSAGYTSSWDFDYW	0.88
Pt42	IGHV3-66	CAKSGGYSSSWDFDYW	0.005571	97.54	IGHV3-30	CAKSAGYTSSWDFDYW	0.88
Pt42	IGHV3-74	CAKSGGYSSSWDFDYW	0.005571	90.97	IGHV3-30	CAKSAGYTSSWDFDYW	0.88
Pt42	IGHV3-11	CAKSGGYSSSWDFDYW	0.003714	97.92	IGHV3-30	CAKSAGYTSSWDFDYW	0.88
Pt42	IGHV3-49	CARDLGGNFDYW	0.003714	100	IGHV3-66	CARDLGGYFDYW	0.92
Pt42	IGHV3-53	CARDLGGNFDYW	0.003714	98.6	IGHV3-66	CARDLGGYFDYW	0.92
Pt42	IGHV3-64	CARDLGGNFDYW	0.003714	100	IGHV3-66	CARDLGGYFDYW	0.92
Pt42	IGHV3-23	CARDLGGNFDYW	0.001857	92.36	IGHV3-66	CARDLGGYFDYW	0.92
Pt42	IGHV3-30	CARDLGGNFDYW	0.072426	98.26	IGHV3-66	CARDLGGYFDYW	0.92
Pt42	IGHV3-43	CARDLGGNFDYW	0.001857	89.24	IGHV3-66	CARDLGGYFDYW	0.92
Pt42	IGHV3-33	CARDLGGNFDYW	0.042713	99.65	IGHV3-66	CARDLGGYFDYW	0.92
Pt42	IGHV3-66	CARDLGGNFDYW	0.001857	96.84	IGHV3-66	CARDLGGYFDYW	0.92
Pt42	IGHV3-NL1	CARDLGGNFDYW	0.001857	92.01	IGHV3-66	CARDLGGYFDYW	0.92
Pt42	IGHV3-30-3	CARDLGGNFDYW	0.027856	96.88	IGHV3-66	CARDLGGYFDYW	0.92
Pt42	IGHV3-11	CARDLGGNFDYW	0.024142	100	IGHV3-66	CARDLGGYFDYW	0.92
Pt42	IGHV3-13	CARDLGGNFDYW	0.016714	97.89	IGHV3-66	CARDLGGYFDYW	0.92
Pt42	IGHV3-21	CARDLGGNFDYW	0.61655	100	IGHV3-66	CARDLGGYFDYW	0.92
Pt42	IGHV3-48	CARDLGGNFDYW	0.013	99.65	IGHV3-66	CARDLGGYFDYW	0.92
Pt42	IGHV3-7	CARDLGGNFDYW	0.009285	100	IGHV3-66	CARDLGGYFDYW	0.92
Pt42	IGHV3-74	CARDLGGNFDYW	0.009285	97.92	IGHV3-66	CARDLGGYFDYW	0.92
Pt42	IGHV3-20	CARDLGGNFDYW	0.005571	91.32	IGHV3-66	CARDLGGYFDYW	0.92
Pt42	IGHV3-30	CARDLGGSFDYW	0.001857	94.1	IGHV3-66	CARDLGGYFDYW	0.92
Pt42	IGHV3-33	CARDLGGSFDYW	0.001857	96.88	IGHV3-66	CARDLGGYFDYW	0.92
Pt42	IGHV3-21	CARDLGGSFDYW	0.003714	100	IGHV3-66	CARDLGGYFDYW	0.92
Pt43	IGHV3-74	CAKSSQAYYYYYYGMDVW	0.001551	95.14	IGHV3-30	CAKSSGSYYYYYYGMDVW	0.89
Pt43	IGHV3-33	CAKSSRAYYYYYYGMDVW	0.007755	100	IGHV3-30	CAKSSGSYYYYYYGMDVW	0.89
Pt43	IGHV3-66	CAKSSRAYYYYYYGMDVW	0.006204	99.65	IGHV3-30	CAKSSGSYYYYYYGMDVW	0.89
Pt43	IGHV3-11	CAKSSRAYYYYYYGMDVW	0.004653	99.65	IGHV3-30	CAKSSGSYYYYYYGMDVW	0.89
Pt43	IGHV3-13	CAKSSRAYYYYYYGMDVW	0.004653	99.3	IGHV3-30	CAKSSGSYYYYYYGMDVW	0.89
Pt43	IGHV3-74	CAKSSRAYYYYYYGMDVW	0.004653	91.67	IGHV3-30	CAKSSGSYYYYYYGMDVW	0.89
Pt43	IGHV3-21	CAKSSRAYYYYYYGMDVW	0.003102	98.26	IGHV3-30	CAKSSGSYYYYYYGMDVW	0.89
Pt43	IGHV3-30	CAKSSRAYYYYYYGMDVW	0.223346	100	IGHV3-30	CAKSSGSYYYYYYGMDVW	0.89
Pt43	IGHV3-7	CAKSSRAYYYYYYGMDVW	0.003102	96.53	IGHV3-30	CAKSSGSYYYYYYGMDVW	0.89
Pt43	IGHV3-15	CAKSSRAYYYYYYGMDVW	0.001551	98.3	IGHV3-30	CAKSSGSYYYYYYGMDVW	0.89
Pt43	IGHV3-30-3	CAKSSRAYYYYYYGMDVW	0.010857	99.65	IGHV3-30	CAKSSGSYYYYYYGMDVW	0.89
Pt43	IGHV3-11	CARANSGSYYMFDYW	0.003102	98.26	IGHV3-30	CARTNSGSYYPFDYW	0.87
Pt43	IGHV3-30	CARANSGSYYMFDYW	0.003102	98.96	IGHV3-30	CARTNSGSYYPFDYW	0.87
Pt43	IGHV3-73	CARANSGSYYMFDYW	0.001551	97.28	IGHV3-30	CARTNSGSYYPFDYW	0.87
Pt43	IGHV3-74	CARANSGSYYMFDYW	0.001551	90.97	IGHV3-30	CARTNSGSYYPFDYW	0.87
Pt43	IGHV3-21	CARANSGSYYMFDYW	0.023265	100	IGHV3-30	CARTNSGSYYPFDYW	0.87
Pt43	IGHV3-11	CARDGVGLYGMDVW	0.006204	99.65	IGHV3-66	CARDLVVLYGMDVW	0.86
Pt43	IGHV3-48	CARDGVGLYGMDVW	0.006204	100	IGHV3-66	CARDLVVLYGMDVW	0.86
Pt43	IGHV3-33	CARDGVGLYGMDVW	0.003102	99.31	IGHV3-66	CARDLVVLYGMDVW	0.86
Pt43	IGHV3-64	CARDGVGLYGMDVW	0.003102	100	IGHV3-66	CARDLVVLYGMDVW	0.86
Pt43	IGHV3-64D	CARDGVGLYGMDVW	0.003102	98.96	IGHV3-66	CARDLVVLYGMDVW	0.86
Pt43	IGHV3-7	CARDGVGLYGMDVW	0.003102	96.53	IGHV3-66	CARDLVVLYGMDVW	0.86
Pt43	IGHV3-74	CARDGVGLYGMDVW	0.003102	99.65	IGHV3-66	CARDLVVLYGMDVW	0.86
Pt43	IGHV3-66	CARDGVGLYGMDVW	0.203183	100	IGHV3-66	CARDLVVLYGMDVW	0.86
Pt43	IGHV3-13	CARDGVGLYGMDVW	0.001551	92.98	IGHV3-66	CARDLVVLYGMDVW	0.86
Pt43	IGHV3-15	CARDGVGLYGMDVW	0.001551	96.26	IGHV3-66	CARDLVVLYGMDVW	0.86
Pt43	IGHV3-30-3	CARDGVGLYGMDVW	0.001551	100	IGHV3-66	CARDLVVLYGMDVW	0.86
Pt43	IGHV3-73	CARDGVGLYGMDVW	0.001551	98.64	IGHV3-66	CARDLVVLYGMDVW	0.86
Pt43	IGHV3-NL1	CARDGVGLYGMDVW	0.001551	96.84	IGHV3-66	CARDLVVLYGMDVW	0.86
Pt43	IGHV3-30	CARDGVGLYGMDVW	0.020163	99.31	IGHV3-66	CARDLVVLYGMDVW	0.86
Pt43	IGHV3-53	CARDGVGLYGMDVW	0.013959	99.3	IGHV3-66	CARDLVVLYGMDVW	0.86

Pt43	IGHV4-39	CARVGPYYYYGMDVW	0.001551	100	IGHV4-34	CARVGGYYYYYMDVW	0.87
Pt44	IGHV3-15	CARDLVGYFDYW	0.002095	90.14	IGHV3-66	CARDLGGYFDYW	0.92
Pt44	IGHV3-48	CARDLVGYFDYW	0.002095	99.65	IGHV3-66	CARDLGGYFDYW	0.92
Pt44	IGHV3-11	CARDLVGYFDYW	0.006285	99.65	IGHV3-66	CARDLGGYFDYW	0.92
Pt44	IGHV3-20	CARDLVGYFDYW	0.00419	98.96	IGHV3-66	CARDLGGYFDYW	0.92
Pt44	IGHV3-30	CARDLVGYFDYW	0.00419	98.61	IGHV3-66	CARDLGGYFDYW	0.92
Pt44	IGHV3-30-3	CARDLVGYFDYW	0.00419	98.61	IGHV3-66	CARDLGGYFDYW	0.92
Pt44	IGHV3-33	CARDLVGYFDYW	0.00419	97.57	IGHV3-66	CARDLGGYFDYW	0.92
Pt44	IGHV3-7	CARDLVGYFDYW	0.354082	100	IGHV3-66	CARDLGGYFDYW	0.92
Pt44	IGHV3-73	CARDLVGYFDYW	0.00419	97.37	IGHV3-66	CARDLGGYFDYW	0.92
Pt44	IGHV3-33	CARDRLDYYGMDVW	0.002095	99.65	IGHV3-53	CARDVGDYYGMDVW	0.86
Pt44	IGHV3-7	CARGLVGYFDYW	0.002095	99.65	IGHV3-66	CARELVGYFDYW	0.92
Pt45	IGHV3-30	CARSVLTYYYFDYW	0.001523	99.31	IGHV3-30-3	CARSVAGYYYFDYW	0.86
Pt47	IGHV3-30	CAKDQDYDFWSGYSNWFDPW	0.003423	96.53	IGHV3-23	CAKDRYYEFWSGYSNWFDPW	0.85
Pt48	IGHV3-30-3	CARSKSGGYSTPFDYW	0.048964	97.57	IGHV3-30-3	CARSSSGGYSAPFDYW	0.88
Pt48	IGHV3-30	CARSKSGGYSTPFDYW	0.012241	97.57	IGHV3-30-3	CARSSSGGYSAPFDYW	0.88
Pt48	IGHV4-61	CARVYYYYYMDVW	0.00408	100	IGHV4-4	CARAYYYYYMDVW	0.92
Pt48	IGHV4-61	CARVYYYYYYMDVW	0.13057	100	IGHV4-30-2	CARVASYYYYMDVW	0.86
Pt48	IGHV4-30-4	CARVYYYYYYMDVW	0.00408	98.28	IGHV4-30-2	CARVASYYYYMDVW	0.86
Pt48	IGHV4-31	CARVYYYYYYMDVW	0.00408	97.93	IGHV4-30-2	CARVASYYYYMDVW	0.86
Pt48	IGHV4-4	CARVYYYYYYMDVW	0.00408	99.65	IGHV4-30-2	CARVASYYYYMDVW	0.86
Pt48	IGHV4-39	CARVYYYYYYMDVW	0.016321	97.59	IGHV4-30-2	CARVASYYYYMDVW	0.86
Pt49	IGHV3-30	CARDLAAAGGFDPW	0.003463	99.65	IGHV3-53	CARDLAAAGAFDIW	0.86
Pt49	IGHV3-33	CARDRGGYFDYW	0.003463	99.65	IGHV3-30-3	CARDGGGYFDYW	0.92
Pt49	IGHV3-7	CARDRGGYFDYW	0.003463	99.31	IGHV3-30-3	CARDGGGYFDYW	0.92
Pt49	IGHV3-30	CARDRGGYFDYW	0.045014	98.61	IGHV3-30-3	CARDGGGYFDYW	0.92
Pt49	IGHV3-48	CARDRGGYFDYW	0.01385	98.61	IGHV3-30-3	CARDGGGYFDYW	0.92
Pt49	IGHV3-11	CARDRGGYFDYW	0.896814	100	IGHV3-30-3	CARDGGGYFDYW	0.92
Pt49	IGHV3-21	CARDRGGYFDYW	0.010388	99.31	IGHV3-30-3	CARDGGGYFDYW	0.92
Pt49	IGHV3-30-3	CARDRGGYFDYW	0.006925	100	IGHV3-30-3	CARDGGGYFDYW	0.92
Pt49	IGHV3-74	CARDRGGYFDYW	0.006925	96.53	IGHV3-30-3	CARDGGGYFDYW	0.92
Pt49	IGHV3-72	CARERGGYFDYW	0.003463	91.16	IGHV3-66	CARERGGRFDYW	0.92
Pt5	IGHV1-2	CARDLGYCSSTSCYYYYYGMDVW	0.002698	100	IGHV1-69	CARDKGYCSSTSCYNYYYGIDVW	0.87
Pt51	IGHV3-48	CARDLGVTTGLFDYW	0.005776	99.65	IGHV3-7	CARDQGVTTGPFDYW	0.87
Pt51	IGHV3-53	CARDLGVTTGLFDYW	0.003851	99.3	IGHV3-7	CARDQGVTTGPFDYW	0.87
Pt51	IGHV3-64	CARDLGVTTGLFDYW	0.003851	98.61	IGHV3-7	CARDQGVTTGPFDYW	0.87
Pt51	IGHV3-66	CARDLGVTTGLFDYW	0.003851	97.89	IGHV3-7	CARDQGVTTGPFDYW	0.87
Pt51	IGHV3-7	CARDLGVTTGLFDYW	0.003851	96.18	IGHV3-7	CARDQGVTTGPFDYW	0.87
Pt51	IGHV3-21	CARDLGVTTGLFDYW	0.001925	94.44	IGHV3-7	CARDQGVTTGPFDYW	0.87
Pt51	IGHV3-30	CARDLGVTTGLFDYW	0.213704	100	IGHV3-7	CARDQGVTTGPFDYW	0.87
Pt51	IGHV3-53	CARGEAGNYYYGMDVW	0.001925	100	IGHV3-30-3	CARGHTGNYYYGMDVW	0.88
Pt51	IGHV3-74	CARGEAGNYYYGMDVW	0.001925	95.14	IGHV3-30-3	CARGHTGNYYYGMDVW	0.88
Pt51	IGHV3-53	CARGEAGNYYYGMEVW	0.001925	100	IGHV3-30	CARGSAGNYYYGMDVW	0.88
Pt51	IGHV3-48	CARGGGGHYYYMDVW	0.005776	99.31	IGHV3-30	CARGFGGNYYYMDVW	0.87
Pt51	IGHV3-74	CARGGGGHYYYMDVW	0.005776	98.26	IGHV3-30	CARGFGGNYYYMDVW	0.87
Pt51	IGHV3-30	CARGGGGHYYYMDVW	0.103964	95.83	IGHV3-30	CARGFGGNYYYMDVW	0.87
Pt51	IGHV3-64	CARGGGGHYYYMDVW	0.003851	98.61	IGHV3-30	CARGFGGNYYYMDVW	0.87
Pt51	IGHV3-66	CARGGGGHYYYMDVW	0.003851	97.89	IGHV3-30	CARGFGGNYYYMDVW	0.87
Pt51	IGHV3-11	CARGGGGHYYYMDVW	0.001925	96.53	IGHV3-30	CARGFGGNYYYMDVW	0.87
Pt51	IGHV3-53	CARGGGGHYYYMDVW	0.001925	83.86	IGHV3-30	CARGFGGNYYYMDVW	0.87
Pt51	IGHV3-7	CARGGGGHYYYMDVW	0.001925	99.31	IGHV3-30	CARGFGGNYYYMDVW	0.87
Pt51	IGHV4-34	CARGQLAYDAFDIW	0.001925	96.13	IGHV4-59	CARGQLRGDAFDIW	0.86
Pt51	IGHV3-21	CARSYSGNYYDAFDIW	0.003851	99.31	IGHV3-30	CAKSYNGNYYDAFDIW	0.88
Pt51	IGHV3-48	CARSYSGNYYDAFDIW	0.003851	98.26	IGHV3-30	CAKSYNGNYYDAFDIW	0.88
Pt51	IGHV3-66	CARSYSGNYYDAFDIW	0.003851	99.65	IGHV3-30	CAKSYNGNYYDAFDIW	0.88
Pt51	IGHV3-74	CARSYSGNYYDAFDIW	0.003851	96.18	IGHV3-30	CAKSYNGNYYDAFDIW	0.88
Pt51	IGHV3-30	CARSYSGNYYDAFDIW	0.080861	98.26	IGHV3-30	CAKSYNGNYYDAFDIW	0.88
Pt51	IGHV3-11	CARSYSGNYYDAFDIW	0.001925	96.18	IGHV3-30	CAKSYNGNYYDAFDIW	0.88
Pt51	IGHV3-53	CARSYSGNYYDAFDIW	0.001925	98.6	IGHV3-30	CAKSYNGNYYDAFDIW	0.88
Pt53	IGHV3-11	CARDGDHYYGMDVW	0.010102	100	IGHV3-53	CARGGGHYYGMDVW	0.86
Pt53	IGHV3-11	CARDGDHYYYAMDVW	0.001122	100	IGHV3-48	CARDGFYYYYAMDVW	0.87
Pt53	IGHV3-11	CARDGDYYYYGMDVW	0.001122	97.22	IGHV3-48	CARDGFYYYYAMDVW	0.87
Pt53	IGHV1-69	CARDTYYYDSSGYHDAFDIW	0.001122	100	IGHV1-69	CARKTYYYDSSGYPEAFDIW	0.85
Pt53	IGHV3-11	CARGGDHYYYGMDVW	0.001122	98.26	IGHV3-30	CARGRDGYYYGMDVW	0.87
Pt53	IGHV3-43	CARGGDHYYYGMDVW	0.001122	96.53	IGHV3-30	CARGRDGYYYGMDVW	0.87
Pt54	IGHV3-74	CARDYGDYGYFDYW	0.008963	97.92	IGHV3-30	CAKDYGDYGAFDYW	0.86
Pt54	IGHV3-11	CARDYGDYGYFDYW	0.004481	98.96	IGHV3-30	CAKDYGDYGAFDYW	0.86
Pt54	IGHV3-15	CARDYGDYGYFDYW	0.004481	92.52	IGHV3-30	CAKDYGDYGAFDYW	0.86
Pt54	IGHV3-20	CARDYGDYGYFDYW	0.004481	94.79	IGHV3-30	CAKDYGDYGAFDYW	0.86
Pt54	IGHV3-72	CARDYGDYGYFDYW	0.004481	95.92	IGHV3-30	CAKDYGDYGAFDYW	0.86
Pt54	IGHV3-33	CARDYGDYGYFDYW	0.03585	100	IGHV3-30	CAKDYGDYGAFDYW	0.86
Pt54	IGHV3-48	CARDYGDYGYFDYW	0.013444	100	IGHV3-30	CAKDYGDYGAFDYW	0.86
Pt54	IGHV3-7	CARDYGDYGYFDYW	0.255434	100	IGHV3-30	CAKDYGDYGAFDYW	0.86
Pt54	IGHV3-30	CARDYGDYGYFDYW	0.008963	98.26	IGHV3-30	CAKDYGDYGAFDYW	0.86
Pt54	IGHV3-7	CARDYGDYGYLDYW	0.004481	100	IGHV3-30	CAKDYGDYGGLDYW	0.86
Pt54	IGHV1-69	CAREGVGATTGFDYW	0.004481	100	IGHV1-46	CAREGVGATAYFDYW	0.87
Pt54	IGHV4-31	CARVLRYYYYYMDVW	0.008963	99.66	IGHV4-34	CARVGGYYYYYMDVW	0.87

Pt54	IGHV4-59	CARVLRYYYYYMDVW	0.008963	99.65	IGHV4-34	CARVGGYYYYYMDVW	0.87
Pt54	IGHV4-39	CARVLRYYYYYMDVW	0.004481	95.53	IGHV4-34	CARVGGYYYYYMDVW	0.87
Pt56	IGHV3-7	CARVDSGYYYYGMDVW	0.003549	100	IGHV3-30	CARADSGSYYYGMDVW	0.88
Pt56	IGHV3-30	CARVDSGYYYYGMDVW	0.001774	99.65	IGHV3-30	CARADSGSYYYGMDVW	0.88
Pt57	IGHV3-21	CARDLDSSSSAFDYW	0.002878	97.22	IGHV3-21	CARDPDSSSSLFDYW	0.87
Pt57	IGHV3-30	CARDLDSSSSAFDYW	0.002878	94.04	IGHV3-21	CARDPDSSSSLFDYW	0.87
Pt57	IGHV3-7	CARDLDSSSSAFDYW	0.002878	93.4	IGHV3-21	CARDPDSSSSLFDYW	0.87
Pt57	IGHV3-33	CARDLDSSSSAFDYW	0.03741	99.65	IGHV3-21	CARDPDSSSSLFDYW	0.87
Pt57	IGHV3-30	CARMDGYNSPFDYW	0.005755	99.65	IGHV3-30	CARGDGYNSPFDYW	0.93
Pt57	IGHV3-11	CARMDGYNSPFDYW	0.002878	93.06	IGHV3-30	CARGDGYNSPFDYW	0.93
Pt57	IGHV3-30-3	CARMDGYNSPFDYW	0.002878	97.92	IGHV3-30	CARGDGYNSPFDYW	0.93
Pt57	IGHV3-74	CARMDGYNSPFDYW	0.002878	94.1	IGHV3-30	CARGDGYNSPFDYW	0.93
Pt59	IGHV3-13	CARGQLGMDVW	0.001655	99.65	IGHV3-20	CARGQGGMDVW	0.91
Pt6	IGHV3-30	CAKGRSLYW	0.00277	100	IGHV3-23	CAPGRSLYW	0.89
Pt6	IGHV3-33	CARDRRVSGGMDVW	0.00554	99.65	IGHV3-53	CARDLDVSGGMDVW	0.86
Pt6	IGHV3-64	CARDRRVSGGMDVW	0.00554	97.57	IGHV3-53	CARDLDVSGGMDVW	0.86
Pt6	IGHV3-74	CARDRRVSGGMDVW	0.00554	98.61	IGHV3-53	CARDLDVSGGMDVW	0.86
Pt6	IGHV3-21	CARDRRVSGGMDVW	0.191115	100	IGHV3-53	CARDLDVSGGMDVW	0.86
Pt6	IGHV3-23	CARDRRVSGGMDVW	0.00277	95.83	IGHV3-53	CARDLDVSGGMDVW	0.86
Pt6	IGHV3-48	CARDRRVSGGMDVW	0.00277	89.58	IGHV3-53	CARDLDVSGGMDVW	0.86
Pt6	IGHV3-53	CARDRRVSGGMDVW	0.00277	97.54	IGHV3-53	CARDLDVSGGMDVW	0.86
Pt6	IGHV3-66	CARDRRVSGGMDVW	0.00277	97.54	IGHV3-53	CARDLDVSGGMDVW	0.86
Pt6	IGHV3-11	CARDRRVSGGMDVW	0.011079	98.96	IGHV3-53	CARDLDVSGGMDVW	0.86
Pt6	IGHV3-30	CARDRRVSGGMDVW	0.011079	100	IGHV3-53	CARDLDVSGGMDVW	0.86
Pt6	IGHV1-3	CAREWGGGSYFDYW	0.022158	100	IGHV1-46	CAREHGGNSYFDYW	0.86
Pt6	IGHV3-13	CARGGAYYYGMDVW	0.00277	98.6	IGHV3-66	CARGAGYYYGMDVW	0.86
Pt6	IGHV3-21	CARGGAYYYYMDVW	0.00554	99.65	IGHV3-53	CARGGPYYYAMDVW	0.86
Pt6	IGHV4-34	CARGGAYYYYYMDVW	0.00277	100	IGHV4-34	CARVGGYYYYYMDVW	0.87
Pt61	IGHV4-59	CARGARSFDYW	0.003779	100	IGHV4-61	CARGAASFDYW	0.91
Pt61	IGHV4-34	CARGARSFDYW	0.001889	100	IGHV4-61	CARGAASFDYW	0.91
Pt61	IGHV4-39	CARGARSFDYW	0.001889	98.97	IGHV4-61	CARGAASFDYW	0.91
Pt61	IGHV3-11	CARGGGDYW	0.003779	100	IGHV3-48	CARAGGDYW	0.89
Pt61	IGHV3-30	CARGGGDYW	0.003779	99.65	IGHV3-48	CARAGGDYW	0.89
Pt61	IGHV3-33	CARGGGDYW	0.001889	97.57	IGHV3-48	CARAGGDYW	0.89
Pt61	IGHV3-43	CARGGGDYW	0.001889	96.53	IGHV3-48	CARAGGDYW	0.89
Pt61	IGHV3-43D	CARGGGDYW	0.001889	96.53	IGHV3-48	CARAGGDYW	0.89
Pt61	IGHV3-21	CARGGGDYW	0.060461	100	IGHV3-48	CARAGGDYW	0.89
Pt61	IGHV3-74	CARGGGDYW	0.001889	97.57	IGHV3-48	CARAGGDYW	0.89
Pt61	IGHV3-48	CARGGGDYW	0.005668	98.26	IGHV3-48	CARAGGDYW	0.89
Pt62	IGHV3-30-3	CARDLIERGMDVW	0.004585	97.92	IGHV3-66	CARDLISRGMDVW	0.92
Pt62	IGHV3-33	CARDLIERGMDVW	0.004585	98.26	IGHV3-66	CARDLISRGMDVW	0.92
Pt62	IGHV3-30	CARDLIERGMDVW	0.013755	99.31	IGHV3-66	CARDLISRGMDVW	0.92
Pt62	IGHV3-11	CARDLIERGMDVW	0.00917	98.96	IGHV3-66	CARDLISRGMDVW	0.92
Pt62	IGHV3-13	CARDLIERGMDVW	0.00917	96.14	IGHV3-66	CARDLISRGMDVW	0.92
Pt62	IGHV3-48	CARDLIERGMDVW	0.261348	100	IGHV3-66	CARDLISRGMDVW	0.92
Pt62	IGHV3-21	CARDRGGYSDYW	0.004585	97.89	IGHV3-53	CARDRGGYPDYW	0.92
Pt63	IGHV3-30	CARARGDYYGMDVW	0.008226	98.61	IGHV3-53	CARDVGDYYGMDVW	0.86
Pt63	IGHV3-66	CARARGDYYGMDVW	0.005484	98.25	IGHV3-53	CARDVGDYYGMDVW	0.86
Pt63	IGHV3-74	CARARGDYYGMDVW	0.005484	95.49	IGHV3-53	CARDVGDYYGMDVW	0.86
Pt63	IGHV3-30-3	CARARGDYYGMDVW	0.087741	96.53	IGHV3-53	CARDVGDYYGMDVW	0.86
Pt63	IGHV3-21	CARARGDYYGMDVW	0.002742	95.83	IGHV3-53	CARDVGDYYGMDVW	0.86
Pt63	IGHV3-33	CARARGDYYGMDVW	0.002742	98.96	IGHV3-53	CARDVGDYYGMDVW	0.86
Pt63	IGHV3-48	CARARGDYYGMDVW	0.002742	94.44	IGHV3-53	CARDVGDYYGMDVW	0.86
Pt63	IGHV3-64	CARARGDYYGMDVW	0.002742	92.71	IGHV3-53	CARDVGDYYGMDVW	0.86
Pt63	IGHV3-73	CARARGDYYGMDVW	0.002742	97.28	IGHV3-53	CARDVGDYYGMDVW	0.86
Pt63	IGHV3-30	CARARGGTYYYGMDVW	0.005484	97.22	IGHV3-30	CAKARGGSYYYGMDVW	0.88
Pt63	IGHV3-66	CARARGGTYYYGMDVW	0.002742	96.14	IGHV3-30	CAKARGGSYYYGMDVW	0.88
Pt63	IGHV3-30-3	CARARGGTYYYGMDVW	0.021935	95.83	IGHV3-30	CAKARGGSYYYGMDVW	0.88
Pt63	IGHV3-11	CARDDLLDYW	0.005484	98.96	IGHV3-30-3	CARDQLLDYW	0.90
Pt63	IGHV3-33	CARDDLLDYW	0.005484	99.65	IGHV3-30-3	CARDQLLDYW	0.90
Pt63	IGHV3-21	CARDDLLDYW	0.068548	100	IGHV3-30-3	CARDQLLDYW	0.90
Pt63	IGHV3-30-3	CARDDLLDYW	0.002742	94.79	IGHV3-30-3	CARDQLLDYW	0.90
Pt63	IGHV3-7	CARDDLLDYW	0.002742	96.53	IGHV3-30-3	CARDQLLDYW	0.90
Pt63	IGHV3-48	CARDDLLDYW	0.010968	99.31	IGHV3-30-3	CARDQLLDYW	0.90
Pt63	IGHV4-59	CAREESYYYYMDVW	0.005484	98.25	IGHV4-30-2	CARVASYYYYMDVW	0.86
Pt63	IGHV4-38-2	CAREESYYYYMDVW	0.049354	97.57	IGHV4-30-2	CARVASYYYYMDVW	0.86
Pt63	IGHV4-34	CAREESYYYYMDVW	0.002742	95.42	IGHV4-30-2	CARVASYYYYMDVW	0.86
Pt63	IGHV4-39	CAREESYYYYMDVW	0.002742	97.94	IGHV4-30-2	CARVASYYYYMDVW	0.86
Pt63	IGHV3-30	CAREVRSSGRAGCFGSW	0.008226	88.19	IGHV3-30	CARESRSSGRAGCFDSW	0.88
Pt63	IGHV3-11	CAREVRSSGRAGCFGSW	0.002742	88.54	IGHV3-30	CARESRSSGRAGCFDSW	0.88
Pt63	IGHV3-33	CAREVSGMDVW	0.008226	99.31	IGHV3-53	CAREVYGMDVW	0.91
Pt63	IGHV3-21	CAREVSGMDVW	0.005484	97.19	IGHV3-53	CAREVYGMDVW	0.91
Pt63	IGHV3-NL1	CAREVSGMDVW	0.005484	97.89	IGHV3-53	CAREVYGMDVW	0.91
Pt63	IGHV3-11	CAREVSGMDVW	0.002742	98.26	IGHV3-53	CAREVYGMDVW	0.91
Pt63	IGHV3-13	CAREVSGMDVW	0.002742	95.09	IGHV3-53	CAREVYGMDVW	0.91
Pt63	IGHV3-48	CAREVSGMDVW	0.002742	96.18	IGHV3-53	CAREVYGMDVW	0.91
Pt63	IGHV3-7	CAREVSGMDVW	0.002742	97.22	IGHV3-53	CAREVYGMDVW	0.91

Pt63	IGHV3-30	CAREVSGMDVW	0.024677	98.26	IGHV3-53	CAREVYGMDVW	0.91
Pt63	IGHV3-53	CAREVSGMDVW	0.139837	100	IGHV3-53	CAREVYGMDVW	0.91
Pt63	IGHV4-30-4	CARVGPRGDYFDYW	0.005484	98.62	IGHV4-39	CARVGGEGDYFDYW	0.86
Pt63	IGHV4-34	CARVGPRGDYFDYW	0.005484	98.24	IGHV4-39	CARVGGEGDYFDYW	0.86
Pt63	IGHV4-61	CARVGPRGDYFDYW	0.005484	98.63	IGHV4-39	CARVGGEGDYFDYW	0.86
Pt63	IGHV4-39	CARVGPRGDYFDYW	0.002742	97.19	IGHV4-39	CARVGGEGDYFDYW	0.86
Pt63	IGHV4-59	CARVGPRGDYFDYW	0.030161	95.79	IGHV4-39	CARVGGEGDYFDYW	0.86
Pt65	IGHV4-59	CAQGFDPW	0.065845	97.54	IGHV4-59	CARGFDPW	0.88
Pt65	IGHV4-61	CAQGFDPW	0.007316	99.66	IGHV4-59	CARGFDPW	0.88
Pt65	IGHV3-48	CARDLIERGMDVW	0.003658	99.65	IGHV3-66	CARDLISRGMDVW	0.92
Pt65	IGHV3-33	CAREDRGAFDIW	0.003658	98.61	IGHV3-53	CARELRGAFDIW	0.92
Pt65	IGHV3-48	CAREDRGAFDIW	0.003658	99.65	IGHV3-53	CARELRGAFDIW	0.92
Pt65	IGHV3-7	CAREDRGAFDIW	0.003658	96.18	IGHV3-53	CARELRGAFDIW	0.92
Pt65	IGHV3-21	CAREDRGAFDIW	0.003658	99.31	IGHV3-53	CARELRGAFDIW	0.92
Pt65	IGHV3-13	CARGYSGSYYYFDYW	0.062187	100	IGHV3-30	CARGLSGNYYYFDYW	0.87
Pt66	IGHV3-13	CAKGIAVAGIFDYW	0.001853	98.6	IGHV3-23	CAKEIAVAGCFDYW	0.86
Pt66	IGHV3-48	CAKGIAVAGIFDYW	0.001853	94.1	IGHV3-23	CAKEIAVAGCFDYW	0.86
Pt66	IGHV3-53	CAKGIAVAGIFDYW	0.001853	99.65	IGHV3-23	CAKEIAVAGCFDYW	0.86
Pt66	IGHV3-7	CAKGIAVAGIFDYW	0.001853	97.22	IGHV3-23	CAKEIAVAGCFDYW	0.86
Pt66	IGHV3-21	CAKGIAVAGIFDYW	0.075955	100	IGHV3-23	CAKEIAVAGCFDYW	0.86
Pt66	IGHV3-11	CAKGIAVAGIFDYW	0.011115	100	IGHV3-23	CAKEIAVAGCFDYW	0.86
Pt66	IGHV3-11	CARDGYHYYGMDVW	0.001853	99.65	IGHV3-53	CARGGGHYYGMDVW	0.86
Pt66	IGHV3-7	CARDITGNYYYGMDVW	0.003705	100	IGHV3-30	CAKDRTGNYYYGMDVW	0.88
Pt66	IGHV3-33	CARDITGNYYYGMDVW	0.001853	98.96	IGHV3-30	CAKDRTGNYYYGMDVW	0.88
Pt66	IGHV3-13	CARDRGDYYGMDVW	0.001853	96.14	IGHV3-53	CARDVGDYYGMDVW	0.93
Pt66	IGHV3-48	CARDRRDYYGMDVW	0.005558	100	IGHV3-53	CARDVGDYYGMDVW	0.86
Pt66	IGHV3-13	CARDRRDYYGMDVW	0.56874	100	IGHV3-53	CARDVGDYYGMDVW	0.86
Pt66	IGHV3-11	CARDRRDYYGMDVW	0.003705	99.65	IGHV3-53	CARDVGDYYGMDVW	0.86
Pt66	IGHV3-30	CARDRRDYYGMDVW	0.003705	100	IGHV3-53	CARDVGDYYGMDVW	0.86
Pt66	IGHV3-53	CARDRRDYYGMDVW	0.003705	99.65	IGHV3-53	CARDVGDYYGMDVW	0.86
Pt66	IGHV3-21	CARDRRDYYGMDVW	0.001853	100	IGHV3-53	CARDVGDYYGMDVW	0.86
Pt66	IGHV3-7	CARDRRDYYGMDVW	0.001853	98.61	IGHV3-53	CARDVGDYYGMDVW	0.86
Pt66	IGHV3-74	CARDRRDYYGMDVW	0.001853	94.44	IGHV3-53	CARDVGDYYGMDVW	0.86
Pt66	IGHV3-33	CARDRRDYYGMDVW	0.012968	99.65	IGHV3-53	CARDVGDYYGMDVW	0.86
Pt66	IGHV1-69	CARDRYCSGGSCYSHYYYYYGMDVW	0.001853	100	IGHV1-18	CAREGYCSGGSCYSGYYYYYGMDVW	0.88
Pt66	IGHV1-2	CARGDYW	0.011115	95.49	IGHV1-2	CARLDYW	0.86
Pt67	IGHV1-18	CARDRGYCSGGSCYSDAFDIW	0.001129	100	IGHV1-3	CARVWGYCSGGSCYVDAFDIW	0.86
Pt67	IGHV1-69	CARDRGYCSGGSCYSDAFDIW	0.040644	100	IGHV1-3	CARVWGYCSGGSCYVDAFDIW	0.86
Pt67	IGHV4-30-4	CARGYYYYYMDVW	0.001129	99.31	IGHV4-4	CARAYYYYYMDVW	0.92
Pt68	IGHV1-18	CARDSSGGYDAFDIW	0.226535	100	IGHV1-46	CARDEVGGYDAFDIW	0.87
Pt68	IGHV3-49	CAREVAARPYYYYYMDVW	0.003381	94.22	IGHV3-48	CARERAARLYYYYYMDVW	0.89
Pt69	IGHV3-13	CARDAGAHDAFDIW	0.212185	100	IGHV3-53	CARIAVAHDAFDIW	0.86
Pt69	IGHV3-53	CARDAGAHDAFDIW	0.004681	96.55	IGHV3-53	CARIAVAHDAFDIW	0.86
Pt69	IGHV3-74	CARDAGAHDAFDIW	0.004681	99.65	IGHV3-53	CARIAVAHDAFDIW	0.86
Pt69	IGHV3-30	CARDAGAHDAFDIW	0.00312	98.26	IGHV3-53	CARIAVAHDAFDIW	0.86
Pt69	IGHV3-66	CARDAGAHDAFDIW	0.00312	94.04	IGHV3-53	CARIAVAHDAFDIW	0.86
Pt69	IGHV3-21	CARDAGAHDAFDIW	0.00156	99.65	IGHV3-53	CARIAVAHDAFDIW	0.86
Pt69	IGHV3-7	CARDAGAHDAFDIW	0.00156	99.65	IGHV3-53	CARIAVAHDAFDIW	0.86
Pt69	IGHV3-33	CARDAGAHDAFDIW	0.009361	99.65	IGHV3-53	CARIAVAHDAFDIW	0.86
Pt69	IGHV3-48	CARDAGAHDAFDIW	0.009361	98.61	IGHV3-53	CARIAVAHDAFDIW	0.86
Pt69	IGHV4-34	CARDTDYGDYFDYW	0.00312	98.94	IGHV4-59	CARGADYGDYFDYW	0.86
Pt69	IGHV4-31	CARDTDYGDYFDYW	0.026523	100	IGHV4-59	CARGADYGDYFDYW	0.86
Pt69	IGHV4-59	CARDTDYGDYFDYW	0.00156	96.14	IGHV4-59	CARGADYGDYFDYW	0.86
Pt69	IGHV4-61	CARDTDYGDYFDYW	0.00156	96.91	IGHV4-59	CARGADYGDYFDYW	0.86
Pt69	IGHV3-33	CARESGSLDYW	0.007801	98.26	IGHV3-20	CARGSGSLDYW	0.91
Pt69	IGHV3-48	CARESGSLDYW	0.004681	98.26	IGHV3-20	CARGSGSLDYW	0.91
Pt69	IGHV3-30	CARESGSLDYW	0.00312	97.22	IGHV3-20	CARGSGSLDYW	0.91
Pt69	IGHV3-66	CARESGSLDYW	0.00312	94.74	IGHV3-20	CARGSGSLDYW	0.91
Pt69	IGHV3-74	CARESGSLDYW	0.095171	96.18	IGHV3-20	CARGSGSLDYW	0.91
Pt69	IGHV3-13	CARESGSLDYW	0.00156	95.79	IGHV3-20	CARGSGSLDYW	0.91
Pt69	IGHV3-21	CARESGSLDYW	0.00156	98.96	IGHV3-20	CARGSGSLDYW	0.91
Pt69	IGHV3-53	CARESGSLDYW	0.00156	93.33	IGHV3-20	CARGSGSLDYW	0.91
Pt69	IGHV3-7	CARESGSLDYW	0.00156	97.57	IGHV3-20	CARGSGSLDYW	0.91
Pt69	IGHV4-34	CARGFDYW	0.007801	95.09	IGHV4-4	CARGFDFW	0.88
Pt69	IGHV4-39	CARGFDYW	0.007801	96.91	IGHV4-4	CARGFDFW	0.88
Pt69	IGHV4-4	CARGFDYW	0.004681	99.31	IGHV4-4	CARGFDFW	0.88
Pt69	IGHV4-31	CARGFDYW	0.00312	97.24	IGHV4-4	CARGFDFW	0.88
Pt69	IGHV4-59	CARGFDYW	0.109213	95.44	IGHV4-4	CARGFDFW	0.88
Pt69	IGHV4-61	CARGFDYW	0.00312	100	IGHV4-4	CARGFDFW	0.88
Pt69	IGHV4-31	CARGFGYW	0.00156	95.53	IGHV4-59	CARGFDYW	0.88
Pt69	IGHV4-31	CARWPAADYYYYMDVW	0.00156	100	IGHV4-59	CARGPAATYYYYMDVW	0.88
Pt69	IGHV4-59	CTRGFDYW	0.00156	95.44	IGHV4-59	CARGFDYW	0.88
Pt7	IGHV3-74	CARDSYGMDVW	0.011301	100	IGHV3-53	CARESYGMDVW	0.91
Pt7	IGHV3-30-3	CARDSYGMDVW	0.008476	100	IGHV3-53	CARESYGMDVW	0.91
Pt7	IGHV3-64	CARDSYGMDVW	0.008476	98.26	IGHV3-53	CARESYGMDVW	0.91
Pt7	IGHV3-13	CARDSYGMDVW	0.005651	100	IGHV3-53	CARESYGMDVW	0.91
Pt7	IGHV3-21	CARDSYGMDVW	0.005651	98.26	IGHV3-53	CARESYGMDVW	0.91

Pt7	IGHV3-48	CARDSYGMDVW	0.005651	99.31	IGHV3-53	CARESYGMDVW	0.91
Pt7	IGHV3-7	CARDSYGMDVW	0.005651	100	IGHV3-53	CARESYGMDVW	0.91
Pt7	IGHV3-43	CARDSYGMDVW	0.002825	96.88	IGHV3-53	CARESYGMDVW	0.91
Pt7	IGHV3-73	CARDSYGMDVW	0.002825	98.64	IGHV3-53	CARESYGMDVW	0.91
Pt7	IGHV3-NL1	CARDSYGMDVW	0.002825	96.49	IGHV3-53	CARESYGMDVW	0.91
Pt7	IGHV3-30	CARDSYGMDVW	0.033904	98.96	IGHV3-53	CARESYGMDVW	0.91
Pt7	IGHV3-53	CARDSYGMDVW	0.223202	99.3	IGHV3-53	CARESYGMDVW	0.91
Pt7	IGHV3-66	CARDSYGMDVW	0.025428	97.19	IGHV3-53	CARESYGMDVW	0.91
Pt7	IGHV3-33	CARDSYGMDVW	0.014127	97.92	IGHV3-53	CARESYGMDVW	0.91
Pt7	IGHV3-11	CARDSYGMDVW	0.011301	100	IGHV3-53	CARESYGMDVW	0.91
Pt70	IGHV3-21	CARDRGGSSDYW	0.001616	99.31	IGHV3-30-3	CARDSGGSSDYW	0.92
Pt71	IGHV4-31	CARGRGYGDYADYW	0.072754	94.16	IGHV4-59	CARGEGYGDYFDYW	0.86
Pt71	IGHV1-69	CARPHCSGGSCYGAFDIW	0.009094	100	IGHV1-58	CAAPHCSGGSCYDAFDIW	0.89
Pt72	IGHV3-33	CAKDLVDYGMDVW	0.003454	98.96	IGHV3-66	CARDLVDYGMDVW	0.92
Pt72	IGHV3-21	CARDLADYGMDVW	0.003454	97.92	IGHV3-66	CARDLAVYGMDVW	0.92
Pt72	IGHV3-21	CARDLVDYGMDAW	0.003454	98.96	IGHV3-66	CARDLVDYGMDVW	0.92
Pt72	IGHV3-30	CARDLVDYGMDVW	0.037997	99.65	IGHV3-53	CARDLIDYGMDVW	0.92
Pt72	IGHV3-48	CARDLVDYGMDVW	0.031088	99.65	IGHV3-53	CARDLIDYGMDVW	0.92
Pt72	IGHV3-33	CARDLVDYGMDVW	0.027634	99.65	IGHV3-53	CARDLIDYGMDVW	0.92
Pt72	IGHV3-11	CARDLVDYGMDVW	0.02418	98.96	IGHV3-53	CARDLIDYGMDVW	0.92
Pt72	IGHV3-21	CARDLVDYGMDVW	0.645941	100	IGHV3-53	CARDLIDYGMDVW	0.92
Pt72	IGHV3-53	CARDLVDYGMDVW	0.006908	97.19	IGHV3-53	CARDLIDYGMDVW	0.92
Pt72	IGHV3-64D	CARDLVDYGMDVW	0.006908	99.31	IGHV3-53	CARDLIDYGMDVW	0.92
Pt72	IGHV3-30-3	CARDLVDYGMDVW	0.003454	97.57	IGHV3-53	CARDLIDYGMDVW	0.92
Pt72	IGHV3-30	CARDLVDYGTDVW	0.003454	98.26	IGHV3-53	CARDLVDYGLDVW	0.92
Pt72	IGHV3-21	CARDLVDYSMDVW	0.003454	100	IGHV3-66	CARDLVDYGMDVW	0.92
Pt76	IGHV3-30-3	CARDQGGMDVW	0.001894	98.26	IGHV3-20	CARGQGGMDVW	0.91
Pt76	IGHV3-33	CARDQGGMDVW	0.001894	100	IGHV3-20	CARGQGGMDVW	0.91
Pt76	IGHV3-48	CARDQGGMDVW	0.001894	98.61	IGHV3-20	CARGQGGMDVW	0.91
Pt76	IGHV4-4	CARDYYGSGRFDYW	0.001894	98.26	IGHV4-4	CARRYYGSGSFDYW	0.86
Pt76	IGHV3-13	CAREPVVPGAGRYYYYGMDVW	0.005681	96.49	IGHV3-30	CARALVVPGAPRYYYYGMDVW	0.86
Pt76	IGHV3-48	CAREPVVPGAGRYYYYGMDVW	0.005681	100	IGHV3-30	CARALVVPGAPRYYYYGMDVW	0.86
Pt76	IGHV3-NL1	CAREPVVPGAGRYYYYGMDVW	0.005681	93.33	IGHV3-30	CARALVVPGAPRYYYYGMDVW	0.86
Pt76	IGHV3-11	CAREPVVPGAGRYYYYGMDVW	0.003787	98.96	IGHV3-30	CARALVVPGAPRYYYYGMDVW	0.86
Pt76	IGHV3-15	CAREPVVPGAGRYYYYGMDVW	0.003787	98.64	IGHV3-30	CARALVVPGAPRYYYYGMDVW	0.86
Pt76	IGHV3-7	CAREPVVPGAGRYYYYGMDVW	0.003787	98.96	IGHV3-30	CARALVVPGAPRYYYYGMDVW	0.86
Pt76	IGHV3-23	CAREPVVPGAGRYYYYGMDVW	0.001894	90.62	IGHV3-30	CARALVVPGAPRYYYYGMDVW	0.86
Pt76	IGHV3-30-3	CAREPVVPGAGRYYYYGMDVW	0.001894	99.31	IGHV3-30	CARALVVPGAPRYYYYGMDVW	0.86
Pt76	IGHV3-66	CAREPVVPGAGRYYYYGMDVW	0.053023	95.44	IGHV3-30	CARALVVPGAPRYYYYGMDVW	0.86
Pt76	IGHV3-53	CAREPVVPGAGRYYYYGMDVW	0.268904	96.49	IGHV3-30	CARALVVPGAPRYYYYGMDVW	0.86
Pt76	IGHV3-74	CAREPVVPGAGRYYYYGMDVW	0.017043	96.88	IGHV3-30	CARALVVPGAPRYYYYGMDVW	0.86
Pt76	IGHV3-30	CAREPVVPGAGRYYYYGMDVW	0.011362	98.61	IGHV3-30	CARALVVPGAPRYYYYGMDVW	0.86
Pt76	IGHV3-33	CAREPVVPGAGRYYYYGMDVW	0.009468	98.26	IGHV3-30	CARALVVPGAPRYYYYGMDVW	0.86
Pt76	IGHV4-34	CARGGDYW	0.003787	99.65	IGHV4-59	CARGFDYW	0.88
Pt76	IGHV4-39	CARGGDYW	0.003787	99.31	IGHV4-59	CARGFDYW	0.88
Pt76	IGHV4-4	CARGGDYW	0.013256	100	IGHV4-59	CARGFDYW	0.88
Pt76	IGHV1-69	CARVEAYDSSGYYYDYW	0.011362	97.57	IGHV1-69D	CARVYYYDSSGYYYDYW	0.88
Pt76	IGHV3-53	CARVPVVPGAGRYYYYGMDVW	0.001894	96.84	IGHV3-30	CARALVVPGAPRYYYYGMDVW	0.86
Pt77	IGHV3-48	CARDLSGYFDYW	0.004716	99.65	IGHV3-66	CARDLGGYFDYW	0.92
Pt77	IGHV3-53	CARDLSGYFDYW	0.004716	97.89	IGHV3-66	CARDLGGYFDYW	0.92
Pt77	IGHV3-74	CARDLSGYFDYW	0.004716	95.83	IGHV3-66	CARDLGGYFDYW	0.92
Pt77	IGHV3-30	CARDLSGYFDYW	0.002358	99.65	IGHV3-66	CARDLGGYFDYW	0.92
Pt77	IGHV3-7	CARDLSGYFDYW	0.056588	100	IGHV3-66	CARDLGGYFDYW	0.92
Pt77	IGHV3-73	CARDLSGYFDYW	0.002358	98.98	IGHV3-66	CARDLGGYFDYW	0.92
Pt77	IGHV3-33	CARDLSGYFDYW	0.011789	100	IGHV3-66	CARDLGGYFDYW	0.92
Pt77	IGHV4-31	CARDRGLVRDAFDIW	0.002358	100	IGHV4-4	CARDRGGVGDAFDIW	0.87
Pt77	IGHV3-33	CARGGPRFYGMDVW	0.002358	94.1	IGHV3-66	CARGGGYFYGMDVW	0.86
Pt78	IGHV1-18	CARDYGDTIDYW	0.005143	100	IGHV1-69	CARDRGDTIDYW	0.92
Pt78	IGHV1-46	CARDYGDTIDYW	0.005143	97.57	IGHV1-69	CARDRGDTIDYW	0.92
Pt78	IGHV3-30	CAREHDFWSGYYPGWFDPW	0.005143	97.22	IGHV3-33	CAREGDFWSGYYTGWFDPW	0.89
Pt79	IGHV3-30	CARDSGARGAFDIW	0.003464	86.81	IGHV3-53	CARDLGSRGAFDIW	0.86
Pt79	IGHV4-34	CARERTGGGWFDPW	0.003464	96.83	IGHV4-4	CARPRQGGGWFDPW	0.86
Pt79	IGHV4-30-2	CARERTGGGWFDPW	0.072745	93.47	IGHV4-4	CARPRQGGGWFDPW	0.86
Pt8	IGHV3-33	CARPSGIAAAGIGYYFDYW	0.024145	100	IGHV3-33	CARPSGIAAAGGGYYFDYW	0.95
Pt8	IGHV3-30	CARPSGIAAAGIGYYFDYW	0.006899	98.96	IGHV3-33	CARPSGIAAAGGGYYFDYW	0.95
Pt8	IGHV3-53	CARVLSDAFDIW	0.010348	99.3	IGHV3-53	CARVVSDAFDIW	0.92
Pt8	IGHV3-33	CARVLSDAFDIW	0.006899	99.31	IGHV3-53	CARVVSDAFDIW	0.92
Pt8	IGHV3-48	CARVLSDAFDIW	0.006899	93.06	IGHV3-53	CARVVSDAFDIW	0.92
Pt8	IGHV3-74	CARVLSDAFDIW	0.006899	95.83	IGHV3-53	CARVVSDAFDIW	0.92
Pt8	IGHV3-21	CARVLSDAFDIW	0.003449	100	IGHV3-53	CARVVSDAFDIW	0.92
Pt8	IGHV3-30	CARVLSDAFDIW	0.003449	95.49	IGHV3-53	CARVVSDAFDIW	0.92
Pt8	IGHV3-7	CARVLSDAFDIW	1.58325	97.57	IGHV3-53	CARVVSDAFDIW	0.92
Pt80	IGHV3-66	CARGGGDYYGMDLW	0.002158	98.6	IGHV3-53	CARGGGHYYGMDVW	0.86
Pt80	IGHV3-30	CARGGGGYYYGMDVW	0.002158	98.61	IGHV3-30-3	CARGFGGNYYGMDVW	0.87
Pt80	IGHV3-30	CARVDGGDYYYGMDVW	0.002158	99.31	IGHV3-66	CARVPLGDYYYGMDVW	0.88
Pt9	IGHV3-11	CARVGWGAFDIW	0.142113	100	IGHV3-23	CAKVGWGAFDIW	0.92

Supplementary Table 3: A list of SARS-specific BcR IG clonotypes resembling known therapeutic antibodies.

Patient ID	IGHV gene	IGH CDR3 (aa)	Freq (%)	DB ID	IGHV gene in db entry	IGH CDR3 (aa) in db entry	Protein + Epitope	Similarity Score	Timepoint
**Pt11**	IGHV3-33	CARDGWYYYYGMDVW	0.001008	**REGN10913**	IGHV3-48	CARDGFYYYYAMDVW	S; RBD	0.87	Post-infection
**Pt16**	IGHV3-33	CARDGSDYGMDVW	0.001477	**REGN11000**	IGHV3-66	CARDGSAYGMDVW	S; RBD	0.92	Post-infection
**Pt20**	IGHV3-30-3	CARDYAAAGTDYW	0.000804	**REGN11010**	IGHV3-66	CARDLAAAGTDYW	S; RBD	0.92	Post-infection
**Pt27**	IGHV3-33	CARDYYYYGMDVW	1.662898	**REGN10971**	IGHV3-53	CARDLYYYGMDVW	S; RBD	0.92	Post-infection
**Pt27**	IGHV3-30	CARDYYYYGMDVW	0.04376	**REGN10971**	IGHV3-53	CARDLYYYGMDVW	S; RBD	0.92	Post-infection
**Pt27**	IGHV3-30-3	CARDYYYYGMDVW	0.02188	**REGN10971**	IGHV3-53	CARDLYYYGMDVW	S; RBD	0.92	Post-infection
**Pt27**	IGHV3-48	CARDYYYYGMDVW	0.014587	**REGN10971**	IGHV3-53	CARDLYYYGMDVW	S; RBD	0.92	Post-infection
**Pt27**	IGHV3-20	CARDYYYYGMDVW	0.007293	**REGN10971**	IGHV3-53	CARDLYYYGMDVW	S; RBD	0.92	Post-infection
**Pt27**	IGHV3-21	CARDYYYYGMDVW	0.007293	**REGN10971**	IGHV3-53	CARDLYYYGMDVW	S; RBD	0.92	Post-infection
**Pt27**	IGHV3-53	CARDYYYYGMDVW	0.007293	**REGN10971**	IGHV3-53	CARDLYYYGMDVW	S; RBD	0.92	Post-infection
**Pt27**	IGHV3-7	CARDYYYYGMDVW	0.007293	**REGN10971**	IGHV3-53	CARDLYYYGMDVW	S; RBD	0.92	Post-infection
**Pt29**	IGHV3-33	CAREYGANYYGMDVW	0.001626	**REGN10982**	IGHV3-66	CARGEGANYYGMDVW	S; RBD	0.87	Post-infection
**Pt29**	IGHV3-33	CARGYGAYYYGMDVW	0.003252	**REGN10982**	IGHV3-66	CARGEGANYYGMDVW	S; RBD	0.87	Post-infection
**Pt36**	IGHV3-53	CARDRGGYFDYW	0.139563	**REGN10915**	IGHV3-66	CARDLGGYFDYW	S; RBD	0.92	Post-infection
**Pt36**	IGHV3-30	CARDRGGYFDYW	0.027455	**REGN10915**	IGHV3-66	CARDLGGYFDYW	S; RBD	0.92	Post-infection
**Pt36**	IGHV3-66	CARDRGGYFDYW	0.020591	**REGN10915**	IGHV3-66	CARDLGGYFDYW	S; RBD	0.92	Post-infection
**Pt36**	IGHV3-33	CARDRGGYFDYW	0.009152	**REGN10915**	IGHV3-66	CARDLGGYFDYW	S; RBD	0.92	Post-infection
**Pt36**	IGHV3-11	CARDRGGYFDYW	0.006864	**REGN10915**	IGHV3-66	CARDLGGYFDYW	S; RBD	0.92	Post-infection
**Pt36**	IGHV3-74	CARDRGGYFDYW	0.006864	**REGN10915**	IGHV3-66	CARDLGGYFDYW	S; RBD	0.92	Post-infection
**Pt36**	IGHV3-NL1	CARDRGGYFDYW	0.004576	**REGN10915**	IGHV3-66	CARDLGGYFDYW	S; RBD	0.92	Post-infection
**Pt36**	IGHV3-13	CARDRGGYFDYW	0.002288	**REGN10915**	IGHV3-66	CARDLGGYFDYW	S; RBD	0.92	Post-infection
**Pt36**	IGHV3-23	CARDRGGYFDYW	0.002288	**REGN10915**	IGHV3-66	CARDLGGYFDYW	S; RBD	0.92	Post-infection
**Pt36**	IGHV3-49	CARDRGGYFDYW	0.002288	**REGN10915**	IGHV3-66	CARDLGGYFDYW	S; RBD	0.92	Post-infection
**Pt42**	IGHV3-21	CARDLGGNFDYW	0.61655	**REGN10915**	IGHV3-66	CARDLGGYFDYW	S; RBD	0.92	Post-infection
**Pt42**	IGHV3-30	CARDLGGNFDYW	0.072426	**REGN10915**	IGHV3-66	CARDLGGYFDYW	S; RBD	0.92	Post-infection
**Pt42**	IGHV3-33	CARDLGGNFDYW	0.042713	**REGN10915**	IGHV3-66	CARDLGGYFDYW	S; RBD	0.92	Post-infection
**Pt42**	IGHV3-30-3	CARDLGGNFDYW	0.027856	**REGN10915**	IGHV3-66	CARDLGGYFDYW	S; RBD	0.92	Post-infection
**Pt42**	IGHV3-11	CARDLGGNFDYW	0.024142	**REGN10915**	IGHV3-66	CARDLGGYFDYW	S; RBD	0.92	Post-infection
**Pt42**	IGHV3-13	CARDLGGNFDYW	0.016714	**REGN10915**	IGHV3-66	CARDLGGYFDYW	S; RBD	0.92	Post-infection
**Pt42**	IGHV3-48	CARDLGGNFDYW	0.013	**REGN10915**	IGHV3-66	CARDLGGYFDYW	S; RBD	0.92	Post-infection
**Pt42**	IGHV3-7	CARDLGGNFDYW	0.009285	**REGN10915**	IGHV3-66	CARDLGGYFDYW	S; RBD	0.92	Post-infection
**Pt42**	IGHV3-74	CARDLGGNFDYW	0.009285	**REGN10915**	IGHV3-66	CARDLGGYFDYW	S; RBD	0.92	Post-infection
**Pt42**	IGHV3-20	CARDLGGNFDYW	0.005571	**REGN10915**	IGHV3-66	CARDLGGYFDYW	S; RBD	0.92	Post-infection
**Pt42**	IGHV3-49	CARDLGGNFDYW	0.003714	**REGN10915**	IGHV3-66	CARDLGGYFDYW	S; RBD	0.92	Post-infection
**Pt42**	IGHV3-53	CARDLGGNFDYW	0.003714	**REGN10915**	IGHV3-66	CARDLGGYFDYW	S; RBD	0.92	Post-infection
**Pt42**	IGHV3-64	CARDLGGNFDYW	0.003714	**REGN10915**	IGHV3-66	CARDLGGYFDYW	S; RBD	0.92	Post-infection
**Pt42**	IGHV3-23	CARDLGGNFDYW	0.001857	**REGN10915**	IGHV3-66	CARDLGGYFDYW	S; RBD	0.92	Post-infection
**Pt42**	IGHV3-43	CARDLGGNFDYW	0.001857	**REGN10915**	IGHV3-66	CARDLGGYFDYW	S; RBD	0.92	Post-infection
**Pt42**	IGHV3-66	CARDLGGNFDYW	0.001857	**REGN10915**	IGHV3-66	CARDLGGYFDYW	S; RBD	0.92	Post-infection
**Pt42**	IGHV3-NL1	CARDLGGNFDYW	0.001857	**REGN10915**	IGHV3-66	CARDLGGYFDYW	S; RBD	0.92	Post-infection
**Pt42**	IGHV3-21	CARDLGGSFDYW	0.003714	**REGN10915**	IGHV3-66	CARDLGGYFDYW	S; RBD	0.92	Post-infection
**Pt42**	IGHV3-30	CARDLGGSFDYW	0.001857	**REGN10915**	IGHV3-66	CARDLGGYFDYW	S; RBD	0.92	Post-infection
**Pt42**	IGHV3-33	CARDLGGSFDYW	0.001857	**REGN10915**	IGHV3-66	CARDLGGYFDYW	S; RBD	0.92	Post-infection
**Pt44**	IGHV3-7	CARDLVGYFDYW	0.354082	**REGN10915**	IGHV3-66	CARDLGGYFDYW	S; RBD	0.92	Post-infection
**Pt44**	IGHV3-11	CARDLVGYFDYW	0.006285	**REGN10915**	IGHV3-66	CARDLGGYFDYW	S; RBD	0.92	Post-infection
**Pt44**	IGHV3-20	CARDLVGYFDYW	0.00419	**REGN10915**	IGHV3-66	CARDLGGYFDYW	S; RBD	0.92	Post-infection
**Pt44**	IGHV3-30	CARDLVGYFDYW	0.00419	**REGN10915**	IGHV3-66	CARDLGGYFDYW	S; RBD	0.92	Post-infection
**Pt44**	IGHV3-30-3	CARDLVGYFDYW	0.00419	**REGN10915**	IGHV3-66	CARDLGGYFDYW	S; RBD	0.92	Post-infection
**Pt44**	IGHV3-33	CARDLVGYFDYW	0.00419	**REGN10915**	IGHV3-66	CARDLGGYFDYW	S; RBD	0.92	Post-infection
**Pt44**	IGHV3-73	CARDLVGYFDYW	0.00419	**REGN10915**	IGHV3-66	CARDLGGYFDYW	S; RBD	0.92	Post-infection
**Pt44**	IGHV3-15	CARDLVGYFDYW	0.002095	**REGN10915**	IGHV3-66	CARDLGGYFDYW	S; RBD	0.92	Post-infection
**Pt44**	IGHV3-48	CARDLVGYFDYW	0.002095	**REGN10915**	IGHV3-66	CARDLGGYFDYW	S; RBD	0.92	Post-infection
**Pt49**	IGHV3-11	CARDRGGYFDYW	0.896814	**REGN10915**	IGHV3-66	CARDLGGYFDYW	S; RBD	0.92	Post-infection
**Pt49**	IGHV3-30	CARDRGGYFDYW	0.045014	**REGN10915**	IGHV3-66	CARDLGGYFDYW	S; RBD	0.92	Post-infection
**Pt49**	IGHV3-48	CARDRGGYFDYW	0.01385	**REGN10915**	IGHV3-66	CARDLGGYFDYW	S; RBD	0.92	Post-infection
**Pt49**	IGHV3-21	CARDRGGYFDYW	0.010388	**REGN10915**	IGHV3-66	CARDLGGYFDYW	S; RBD	0.92	Post-infection
**Pt49**	IGHV3-30-3	CARDRGGYFDYW	0.006925	**REGN10915**	IGHV3-66	CARDLGGYFDYW	S; RBD	0.92	Post-infection

**Pt49**	IGHV3-74	CARDRGGYFDYW	0.006925	**REGN10915**	IGHV3-66	CARDLGGYFDYW	S; RBD	0.92	Post-infection
**Pt49**	IGHV3-33	CARDRGGYFDYW	0.003463	**REGN10915**	IGHV3-66	CARDLGGYFDYW	S; RBD	0.92	Post-infection
**Pt49**	IGHV3-7	CARDRGGYFDYW	0.003463	**REGN10915**	IGHV3-66	CARDLGGYFDYW	S; RBD	0.92	Post-infection
**Pt53**	IGHV3-11	CARDGDHYYYAMDVW	0.001122	**REGN10913**	IGHV3-48	CARDGFYYYYAMDVW	S; RBD	0.87	Post-infection
**Pt53**	IGHV3-11	CARDGDYYYYGMDVW	0.001122	**REGN10913**	IGHV3-48	CARDGFYYYYAMDVW	S; RBD	0.87	Post-infection
**Pt77**	IGHV3-7	CARDLSGYFDYW	0.056588	**REGN10915**	IGHV3-66	CARDLGGYFDYW	S; RBD	0.92	Post-infection
**Pt77**	IGHV3-33	CARDLSGYFDYW	0.011789	**REGN10915**	IGHV3-66	CARDLGGYFDYW	S; RBD	0.92	Post-infection
**Pt77**	IGHV3-48	CARDLSGYFDYW	0.004716	**REGN10915**	IGHV3-66	CARDLGGYFDYW	S; RBD	0.92	Post-infection
**Pt77**	IGHV3-53	CARDLSGYFDYW	0.004716	**REGN10915**	IGHV3-66	CARDLGGYFDYW	S; RBD	0.92	Post-infection
**Pt77**	IGHV3-74	CARDLSGYFDYW	0.004716	**REGN10915**	IGHV3-66	CARDLGGYFDYW	S; RBD	0.92	Post-infection
**Pt77**	IGHV3-30	CARDLSGYFDYW	0.002358	**REGN10915**	IGHV3-66	CARDLGGYFDYW	S; RBD	0.92	Post-infection
**Pt77**	IGHV3-73	CARDLSGYFDYW	0.002358	**REGN10915**	IGHV3-66	CARDLGGYFDYW	S; RBD	0.92	Post-infection
**Pt80**	IGHV3-30	CARVDGGDYYYGMDVW	0.002158	**REGN10935**	IGHV3-66	CARVPLGDYYYGMDVW	S; RBD	0.88	Post-infection
**Pt10**	IGHV3-33	CARDYYYYGMDVW	0.00214	**REGN10971**	IGHV3-53	CARDLYYYGMDVW	S; RBD	0.92	Post-vaccination
**Pt54**	IGHV3-33	CARDGSYYYYYMDVW	0.001579	**REGN10913**	IGHV3-48	CARDGFYYYYAMDVW	S; RBD	0.87	Post-vaccination
**Pt10**	IGHV3-11	CARGDGAYYYGMDVW	0.00214	**REGN10982**	IGHV3-66	CARGEGANYYGMDVW	S; RBD	0.87	Post-vaccination
**Pt60**	IGHV1-46	CARDPHYYDSSGYYLDYW	0.006405	**REGN10977**	IGHV1-69	CARTPFYYDSSGYYLDYW	S; RBD	0.89	Post-vaccination
**Pt60**	IGHV1-46	CARGPHYYDSSGYYLDYW	0.002135	**REGN10977**	IGHV1-69	CARTPFYYDSSGYYLDYW	S; RBD	0.89	Post-vaccination
